# Timing of First Systemic Antibiotic Administration and Risk of Deep Infection in Adults with Open Long-Bone Fractures: A Systematic Review and Threshold-Specific Evidence Synthesis

**DOI:** 10.3390/jcm15197689

**Published:** 2026-10-04

**Authors:** Nguyen The Diep, Nguyen Minh Chau, Phan Thanh Nam, Tien Van Nguyen

**Affiliations:** 1 Department of Traumatology, Thai Binh University of Medicine and Pharmacy, Hung Yen 17000, Vietnam; diepnguyentheytb@gmail.com (N.T.D.); nguyenminhcauytb@gmail.com (N.M.C.); phanthanhnamytb@gmail.com (P.T.N.); 2 Department of Healthcare Organisation and Management, Faculty of Public Health, Thai Binh University of Medicine and Pharmacy, Hung Yen 17000, Vietnam

**Keywords:** open fracture, long-bone fracture, antibiotic prophylaxis, fracture-related infection, deep surgical-site infection, treatment delay, systematic review, evidence synthesis

## Abstract

**Background/Objectives:** Early systemic antibiotics are central to open-fracture care, but a 60 min operational target does not establish a biological infection threshold. We examined how time origin, cutoff, and outcome definition affect interpretation of reported associations. **Methods:** Evidence was reconstructed from two preserved bibliographic inventories containing 24 entries. Complete database-search exports were unavailable, and selection, extraction, ROBINS-E, and GRADE were not independently duplicated. The protocol was archived retrospectively on OSF (doi:10.17605/OSF.IO/7J8QN). Estimates were described separately by time origin, cutoff, adjustment, outcome, and follow-up for a target population aged at least 18 years. No pooled estimate was retained. **Results:** The historical evidence map contained 16 reports, including contextual reports that did not establish applicability to the target population. This is not a verified final included-study count. Within the preserved inventory, no threshold-by-time-origin group supplied two compatible adjusted estimates. A 191-patient cohort reported an adjusted odds ratio of 1.02 (95% CI 0.39–2.68) at 60 min after emergency-department arrival. Recorded appraisals indicated predominantly high or very high risk of bias and very low certainty; these judgements were not independently verified. **Conclusions:** Distinguishing care-process targets from causal thresholds improves interpretation of the available reports. However, incomplete searching and single-investigator assessment limit the reliability and completeness of this synthesis. The observational associations establish neither causal thresholds nor the safety of delay and do not justify postponing indicated prophylaxis.

## 1. Introduction

Open fractures create direct communication between the fracture haematoma and the external environment, combining skeletal injury with contamination, soft-tissue disruption, and impaired local perfusion. They most often involve the limbs, particularly the tibia because of its limited soft-tissue envelope. Deep infection can necessitate repeated debridement, prolonged antimicrobial therapy, delayed union or nonunion, flap revision, amputation, and long-term functional loss, while imposing substantial health-system costs [1,2,3,4,5].

As an estimate of total acute inpatient expenditure, Pley et al. reported a median direct cost of £20,398 per patient among 58 patients older than 65 years with open lower-limb fractures treated at one UK centre between March 2014 and March 2019 [6]. This is a setting-specific inpatient cost per patient, not a pooled cost for the timing studies, an all-adult average, or the incremental cost of infection or delayed antibiotics. No cost-effectiveness analysis was performed in this review.

Systemic antibiotic prophylaxis can be initiated immediately once an open fracture is recognised. Randomised evidence supports antibiotics compared with no antibiotics, and contemporary orthopaedic trauma guidelines recommend administration of an appropriate intravenous regimen as early as feasible [7,8,9]. Many trauma systems use administration within 60 min of emergency-department arrival as a quality metric because the interval is measurable and amenable to protocolised improvement.

Whitehouse et al. (2017) identified eight timing studies with substantial risk of bias [10]; the Cochrane review by Chan et al. (2022) found no randomised trial directly comparing the timing of the first antibiotic dose [11] and Marchiori and Nunes (2024) included 14 heterogeneous studies without meta-analysis [12]. The present synthesis separates time origins, numerical cutoffs, adjustment status, deep-infection definitions, and surveillance periods at the study-level. This allows clinicians to distinguish a facility-response measure from total injury-to-treatment delay and to identify which estimates address the same clinical question. Compatible evidence within individual timing comparisons is limited; this is not a claim that the worldwide literature is small. Although the preserved corpus contains later reports, the missing native search records preclude describing it as a verified comprehensive update of those reviews.

The definition of time zero is critical. Injury-to-antibiotic time includes prehospital and transfer delays; first-medical-contact time reflects emergency medical services performance; first-hospital-arrival time measures the response of the initial treating facility; and receiving-trauma-centre time may omit care delivered before transfer. Consequently, identical numerical delays can represent different clinical processes. Pooling these intervals risks exposure misclassification and produces an effect estimate without a coherent clinical interpretation.

Outcome definition and follow-up also vary. Studies have used superficial wound changes, deep surgical-site infection, unplanned irrigation and debridement, osteomyelitis, or composites that include nonunion. Consensus fracture-related infection (FRI) criteria provide a more reproducible framework, and 90-day surveillance may miss clinically important late infections [3,4,5]. Confounding by indication is an additional concern: severely contaminated or vascularly compromised fractures may receive antibiotics sooner because their urgency is recognised, yet, the same injuries have a higher baseline risk of infection.

This review examined study-specific associations between the first systemic antibiotic timing and deep infection or FRI. The one-hour question concerns interpretation of a care-process target: an auditable target does not, by itself, demonstrate that crossing 60 min causes an abrupt increase in infection risk. A causal threshold would require a defined time origin and comparable injury, treatment, and outcome conditions. Accordingly, 60, 120, and 180 min comparisons were described separately, with adjusted estimates prioritised for interpretation. Neither dichotomised observational comparisons nor covariate adjustment can establish the existence or absence of a universal biological change point. The question is not whether indicated prophylaxis can safely be postponed.

## 2. Materials and Methods

### 2.1. Study Design, Reporting Standards, and Registration

Reporting was organised using PRISMA 2020, MOOSE, and relevant PRISMA-S items [13,14,15]. The available materials support a retrospective reconstruction of preserved evidence, not a fully reproducible search across all planned databases. Study-specific estimates were retained without pooling clinically or methodologically incompatible results [16]. The accompanying Supplementary File supplementary distinguishes documented source checks from missing search and independent-assessment records. Use of a reporting framework is not presented as proof that the two unresolved research-conduct requirements were fulfilled.

The review was not prospectively registered. The 5 August 2026 date in earlier versions refers to historical evidence verification; 6 August 2026 is the subsequent date of the retrospective OSF Open-Ended Registration (doi:10.17605/OSF.IO/7J8QN; associated project: https://osf.io/g3vma (accessed on 30 September 2026) [17]. These dates describe different activities, not two competing dates for the same search. Neither establishes a verified last-search date for every database. Supplementary File supplementary, Section SA, records the chronology and later amendments. The archive does not demonstrate that analytical decisions preceded knowledge of the results. The original registration title is preserved in its citation, while the revised manuscript title reflects the withdrawal of the 180 min pooled estimate.

### 2.2. Eligibility Criteria

Population (P): The target population was adults aged at least 18 years with acute open fractures of the humerus, radius, ulna, femur, tibia, or fibula. This age criterion was retained from the submitted manuscript and was not changed to 16 years. Reports including 16- or 17-year-olds, or younger adolescents, could inform the primary adult comparison only through separately reported data for participants aged at least 18 years who also met the other eligibility criteria. Unseparated mixed-age cohorts were described as contextual evidence. Skeletal maturity, treatment in an adult service, and an adult mean age did not establish an adult-only sample. The same separation rule applied to mixed anatomy or ballistic and nonballistic mechanisms. An unspecified age range was recorded as unresolved, rather than assumed eligible or excluded solely because an adult denominator was unreported. Supplementary File supplementary, Sections SC and SK, distinguishes historical retention, primary applicability, and unresolved classification.

Exposure (E): Exposure (E): Elapsed time from the reported starting point injury, first medical contact, first-hospital arrival, or receiving-centre arrival to the true first systemic antibacterial dose intended as prophylaxis for the acute open fracture, including any documented prehospital or pretransfer dose when reported. Comparator (C): Earlier administration within the same cohort and using the same time origin, with the primary report’s reference category retained; the later-treated group was compared with that reference. For a continuous-time model, the contrast was the reported increase in time in its original units, rather than an invented binary group. The numerical categories were 60, 120, and 180 min, with other/data-derived and longer-delay contrasts kept separate. The exact <, ≤, >, or ≥ boundary and reference category were preserved; equality at a cutoff was not inferred when unspecified. These are retrospectively documented analytical categories, not prospectively prespecified thresholds.

Eligible antibiotic exposure was systemic antibacterial prophylaxis started for the acute open fracture, whether a single agent, a combination, or an alternative systemic agent used by the primary study. Intravenous cefazolin-based prophylaxis was included but was not a mandatory universal regimen. Eligibility did not impose a common drug, dose, spectrum, duration, or retrospective definition of guideline concordance. Drug, route, dose, additional coverage, prior doses, and protocol-period changes were recorded when available; missing details were left unreported. Local/topical antibiotics alone, treatment started for an established infection, and comparisons of duration or agent without a first-dose timing contrast did not meet the exposure definition. Concomitant local antibiotics did not erase an otherwise relevant systemic-timing comparison but required separate description.

The primary clinical outcome was deep infection or FRI. Timing comparisons could arise from randomised trials, cohort studies, case-control studies, registry analyses, or controlled before–after studies. Reports without a timing comparison, reviews, editorials, nonhuman studies, and reports limited to digital, axial, pathological, periprosthetic, or inseparable ballistic injuries were not eligible for the primary clinical comparison. Process-improvement, mixed-age, and composite-outcome reports could be described in the contextual inventory but were not treated as equivalent threshold evidence.

### 2.3. Information Sources and Search Strategy

Database-specific strategies were prepared for MEDLINE/PubMed, Embase, Scopus, Web of Science Core Collection, CENTRAL, CINAHL, Global Index Medicus, ClinicalTrials.gov, and WHO ICTRP, with backward and forward citation searching planned. The prepared strategies have no publication-year/date restriction and were intended to cover each source from inception to its actual search date. They also contain no language or age restriction. The year of the oldest retained report is not a search limit. However, without a successful full search log, applied interface filters and completed search dates cannot be certified. Verbatim prepared strings and source-specific execution status are supplied in Supplementary Search Strategies and Supplementary File supplementary, Section SB. Europe PMC remains supplementary rather than a substitute for an unsearched planned source.

The two preserved CSV inventories were reconciled retrospectively without overwriting their original rows: the downloaded-library manifest contains 11 entries and the core-study retrieval log 13. Source-file name, row number, and stable record ID preserve the origin of each entry. Separate working fields standardised the whitespace, author/year information, and DOI/title text for matching; original strings were retained. Candidate identifier matches were checked against the title, authors, year, and publication type. Duplicate citations to the same report were distinguished from an abstract, poster, correction, or full article describing the same study. For example, the Haile poster’s citation of the full article DOI did not justify deleting the poster as a duplicate citation. Kock and Haile companion relationships were recorded separately; the Shannon relationship remained unresolved. Historical dispositions were then mapped to report and study identifiers without inventing retrieval outcomes. The record-level crosswalk and rules are in Supplementary File supplementary, Section SK. This process reconciles the available inventories, not missing database exports or a complete study-selection flow.

Complete source-specific search dates, yields, native exports, and a database-export deduplication trail were unavailable. The preserved revision records document unsuccessful programmatic requests and targeted article checks; the source-verification note dated 22 September 2026 likewise records failed public-source requests rather than a completed search update. A prepared query or an unsuccessful request was not counted as a searched source, and missing yields were not coded as zero. The current synthesis therefore does not claim a comprehensive inception-to-date update or a final database-based PRISMA flow. The source status and its consequences for interpretation are reported in Supplementary File supplementary, Sections SA–SC.

### 2.4. Study Selection Process

**Figure 1:**
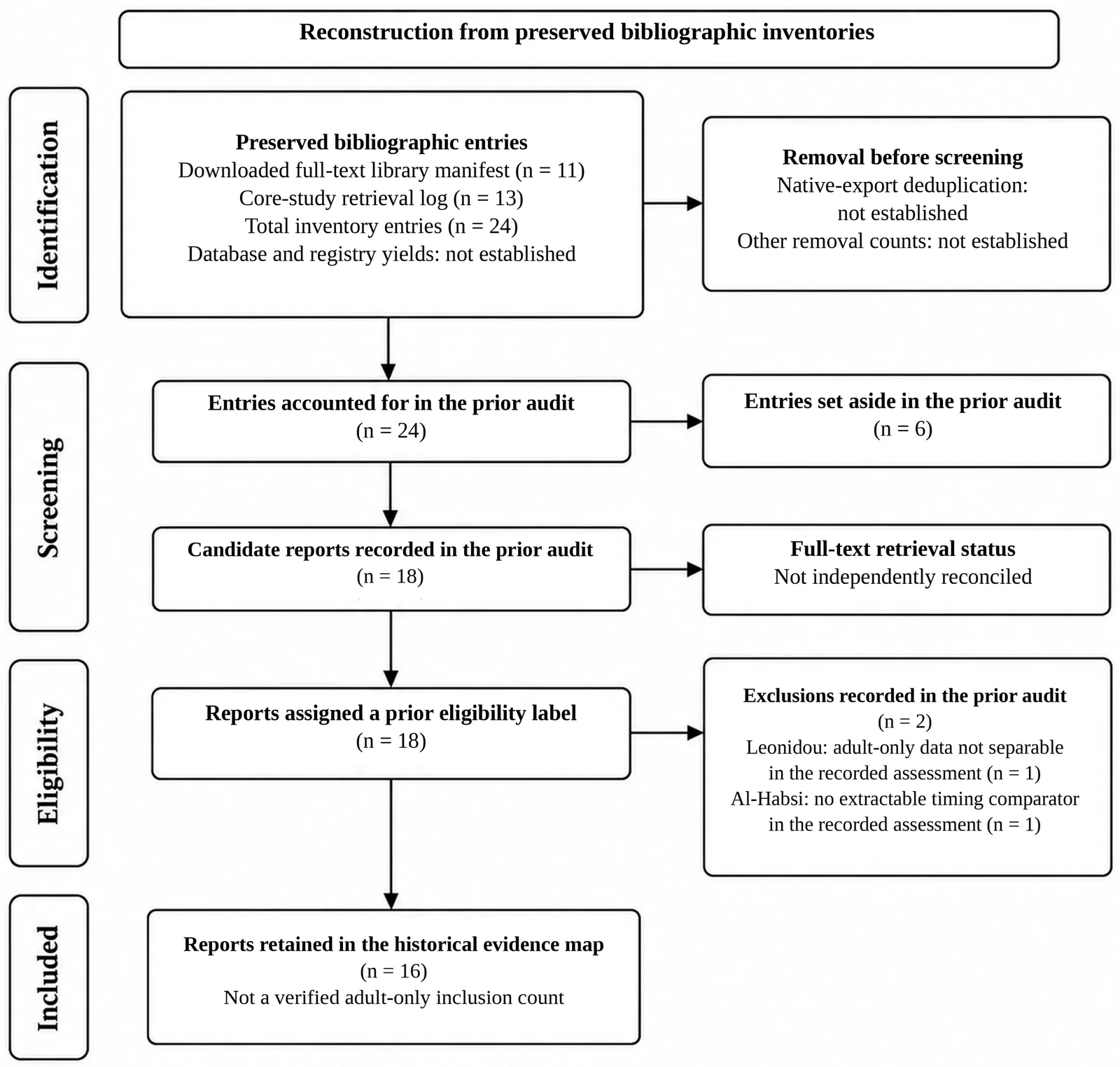
PRISMA 2020-adapted reconstruction of the preserved evidence inventory [13]. Counts derive from the downloaded-library manifest (11 entries) and core-study retrieval log (13 entries). The 24 → 18 → 16 sequence reproduces prior classifications, not a completed database search or independently verified eligibility decisions. Missing source yields, removal counts, and full-text retrieval outcomes are not coded as zero. The final box denotes reports retained in the historical map, not a verified count of eligible adult-only studies. Additional targeted-search candidates are outside this diagram.

The documented original selection was undertaken by one investigator (T.V.N.). The subsequent same-investigator checks compared bibliographic identifiers, citation-versus-companion relationships, and historical eligibility labels with accessible source reports. These checks could identify selected inconsistencies but did not establish independent eligibility decisions or search completeness. Independent final eligibility assessment by two researchers, as described in the Cochrane Handbook [16], was not completed. Figure 1 is consequently labelled as a reconstruction of the historical inventory rather than a verified final study-selection flow. Supplementary File supplementary, Sections SC, SD and SK, specify the scope of the documented checks.

No locked paired eligibility decisions, independently extracted numerical records, or independent ROBINS-E/GRADE assessments were available. Accordingly, no inter-reviewer agreement statistic, screening accuracy, or audit error rate is reported. Arithmetic and internal-consistency checks are distinguished from independent human verification. The four-author byline does not establish that a second researcher performed these procedures, and AI-assisted checks are not counted as an additional reviewer. These limitations apply to the entire evidence synthesis, not only to the former pooled analysis.

Conference abstracts and full reports were linked when the source records supported a companion relationship. Companion reports were not treated as independent samples, and topical similarity alone was not used to declare duplication. Unresolved links and the distinction between report retrieval and distribution status are recorded in Supplementary File supplementary, Section SC.

### 2.5. Data Extraction and Author Queries

The available research materials comprise CSV bibliographic inventories and a structured Excel extraction workbook; no additional historical database or reference-management software was inferred. Each effect entry represents a study–threshold–time-origin–outcome comparison and records design, setting, recruitment period, patients versus fractures, anatomy/severity, transfers, prior antibiotics, regimen, outcome, follow-up, effect measure, precision, covariates, missing data, and possible overlap. The revision files were kept as unchanged source copies and separate versioned working tables. Stable IDs, source page/table or URL, and SHA-256 hashes link changes to their inputs. Cleaning checked identifier consistency, numeric types, group totals, reference direction, units, and the distinction between the minimum, median, and scheduled follow-up. Missing values were retained as missing, not replaced by zero; published and computed values were stored separately. This describes the preserved files and current revision workflow, not an undocumented historical storage system. The local files have not been represented as uploaded to OSF.

Published corrections were linked to the original reports and were not counted as independent studies. Zuelzer et al. was retained because it directly examined first-dose timing and deep infection in open tibia fractures managed with primary closure [18], not because of statistical significance or to increase the evidence count. Its six-week minimum follow-up restricts it to contextual evidence for the primary minimum-90-day question. The linked erratum clarifies the cohort and analysis denominator [18]; it is not a retraction or a replacement timing analysis. The original report and correction are read together as reports of one study. Supplementary File supplementary, Section SE.7, records their bibliographic relationship and the limits of the reported estimate.

**Figure 2:**
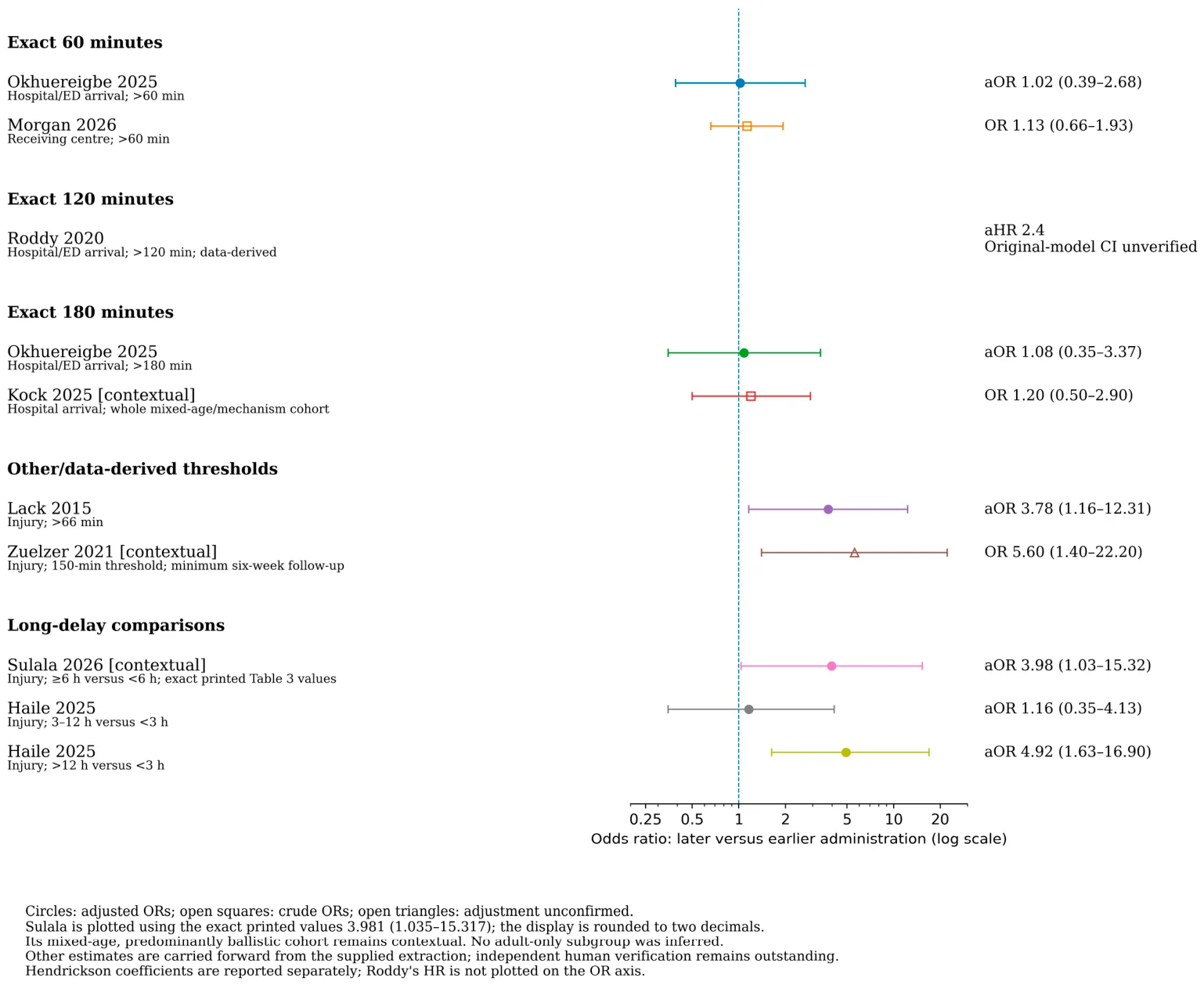
Individual timing estimates without pooling. Markers distinguish adjusted, crude/derived, and unconfirmed-adjustment ORs; equal marker sizes are not weights. The dashed vertical line indicates the null value (OR = 1.0). The plotted estimates are derived from Okhuereigbe et al. [23], Morgan et al. [19], Roddy et al. [24], Kock et al. [20], Lack et al. [25], Zuelzer et al. [18], Sulala et al. [22], and Haile et al. [26]. Kock [20] and Sulala [22] are contextual mixed-age, mixed-mechanism cohorts; Zuelzer [18] is contextual for the minimum-90-day question. Hendrickson’s coefficients are reported on their original scale in Table 1 of the present manuscript, not on the OR axis [21]. Sulala is plotted from the exact values reported in Table 3 of Sulala et al. [22], 3.981 (1.035–15.317), and labelled 3.98 (1.04–15.32). Its ≥6 h versus <6 h boundary is retained. Roddy’s abstract HR is presented textually because the original-model CI remains unverified [24]. No pooled effect is shown. Independent human verification of the extracted evidence was not completed.

The documented extraction checks covered only fields with an available report or supporting passage. Patient and fracture denominators were distinguished; event and non-event totals and the later-versus-earlier reference direction were checked; and adjusted ORs, crude ORs, HRs, and non-ratio coefficients were kept separate. Where source text was available, the stated time origin, pretransfer doses, cutoff operators, outcome definition, and minimum versus scheduled follow-up were compared with the extraction. Morgan [19] and Kock [20] calculations checked whole-cohort arithmetic only, without creating subgroup data. Unresolved ages, model attribution, exact-boundary rules, and original confidence limits remained labelled as such. No primary-study author response was incorporated and no confidence limits were inferred from rounded HRs or *p* values. Supplementary File supplementary, Section SD, separates documented checks from unverified fields; the related source-specific findings and checking calculations are provided in Sections SE and SK. Correct reproduction of selected calculations does not establish that every relevant result was extracted or correctly classified. The 22 September source re-extraction recovered the confidence intervals printed in Hendrickson’s Table 2 [21] and the exact adjusted estimate and six-hour boundary in Sulala’s Tables 2 and 3 [22]. It also identified inconsistencies in Morgan’s anatomical subgroup counts [19] and Sulala’s printed crude-odds-ratio row [22]. Published values and arithmetic checks are retained in Supplementary File supplementary, Sections SE and SK; no author clarification or independent human verification was obtained. Individual timing estimates are presented without pooling in Figure 2.

### 2.6. Classification of Time Origin and Exposure Threshold

Four time origins were kept separate: injury, first medical contact, first-hospital arrival, and receiving-trauma-centre arrival. A reference to emergency-department arrival alone did not establish that the department was the first treating hospital. Accordingly, entries with unresolved first-versus-receiving-facility status are labelled as hospital/ED arrival with the facility status unconfirmed. For transfers, the antibiotics given before arrival at the receiving centre remain the true first dose. A subsequent receiving-centre dose must not reset that endpoint. The four intervals and this transfer-related distinction are illustrated in Supplementary Figure S1.

The 60, 120, and 180 min groups were retained as descriptive categories. Membership in an exact numerical category did not imply that a cutoff had been externally validated or selected before the primary study analysed its data. For example, a 120 min ROC-derived cutoff remains data-derived despite coinciding numerically with two hours. Other/data-derived cutoffs and long-delay comparisons were displayed separately. Continuous-time results were described in their reported units; rounded per-minute estimates were not exponentiated to create unreported hourly effects.

Age eligibility and treatment-delay cutoffs were distinct: the population threshold was at least 18 years, whereas exposure contrasts concerned minutes or hours to the first dose. The full reported group boundary was required before classifying treatment at exactly 60, 120, or 180 min. A <3 h versus >3 h report was not converted to a ≤3 h comparison without knowing how equality was handled.

### 2.7. Causal Framework and Confounding Control

The causal framework distinguishes the total effect of delaying prophylactic antibiotics from the associations available in observational reports. Potential baseline confounders include fracture severity, anatomical site, contamination, vascular injury, overall injury burden, transfer pathways, host susceptibility, and trauma-system capability. These variables can influence both the urgency of antibiotic delivery and infection risk. The framework was documented retrospectively and used to interpret, not eliminate, residual confounding.

Debridement, fixation, and soft-tissue coverage were considered separately from baseline prognostic factors. Their occurrence after the first dose does not by itself establish mediation or make adjustment inappropriate. Depending on the causal structure, conditioning on subsequent care may remove part of a total effect or introduce selection bias. Adjustment sets were therefore described explicitly rather than ranked by the number of covariates. The retrospectively documented conceptual framework and its assumptions are provided in Supplementary File supplementary, Section SH.

### 2.8. Outcome Harmonisation and Follow-Up

The primary outcome hierarchy was: (1) FRI meeting international consensus confirmatory criteria; (2) CDC/NHSN or equivalent deep surgical-site infection; (3) deep infection requiring unplanned irrigation and debridement, implant exchange or removal, or amputation; and (4) clinically, microbiologically, or radiographically confirmed osteomyelitis [3,4]. Superficial infection, cellulitis, drainage without deep involvement, and inseparable superficial/deep composites did not contribute to the primary clinical comparison.

Deep infection or FRI assessed for at least 90 days was prioritised. The longer-term question required both a minimum of 12 months of ascertainment and an explicit outcome definition. Scheduled 12-month visits were not treated as proof that every participant completed 12 months, and the follow-up to union was not assumed to equal a fixed minimum duration. Studies with shorter or unclear surveillance remained in the contextual inventory. These restrictions informed interpretation; they did not produce a new pooled sensitivity analysis.

### 2.9. Risk of Bias Assessment

**Figure 3:**
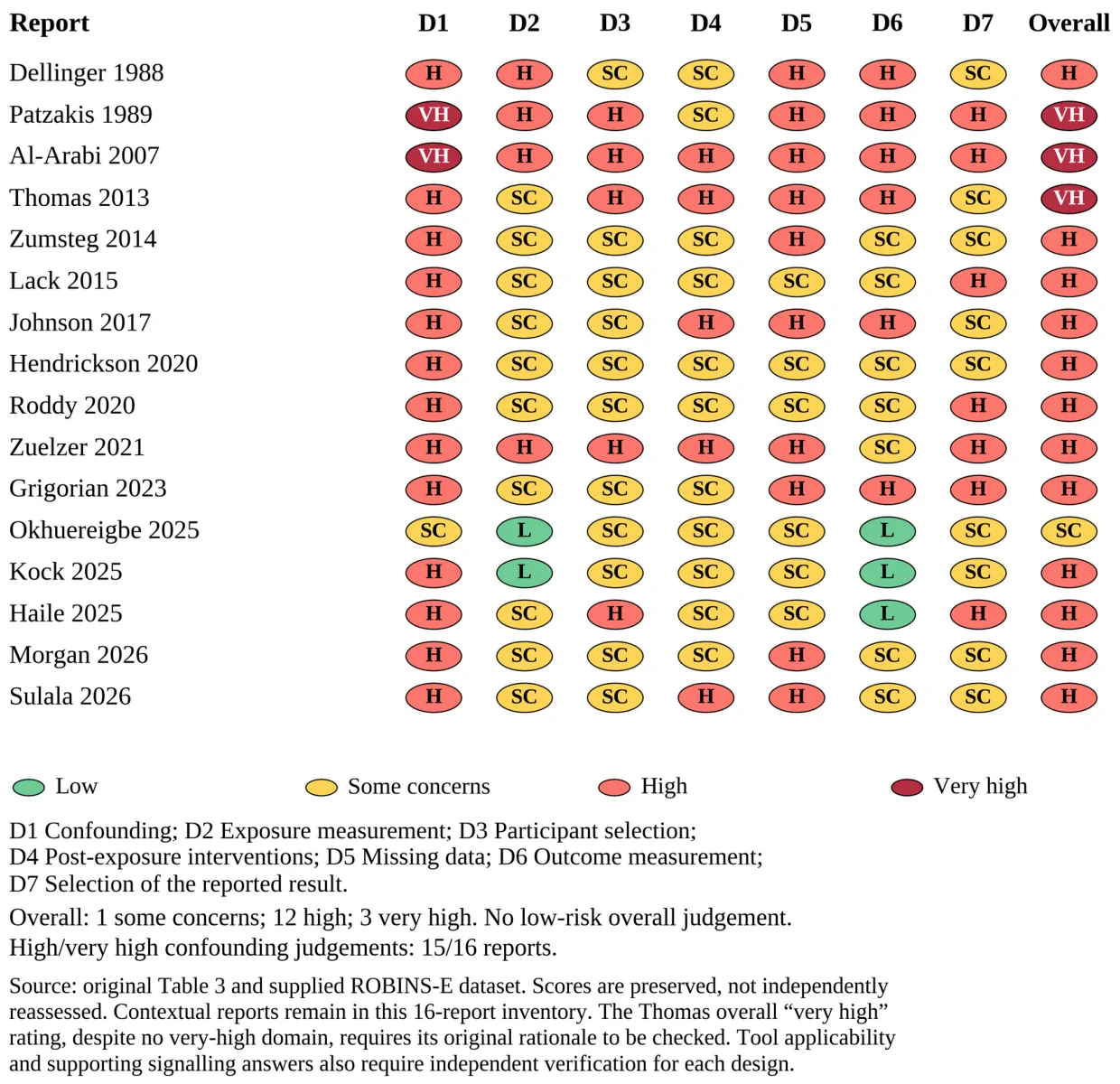
Historical ROBINS-E domain and overall judgements. The matrix reproduces the recorded single-investigator assessments for the reports shown in the figure [18,19,20,21,22,23,24,25,26,28,29,30,31,32,33,34], using the ROBINS-E framework [27]. The recorded totals are one report with some concerns, 12 with high risk, and three with very high risk. Thomas’s overall very-high judgement is a possible cumulative override under ROBINS-E, not an automatic scoring error. Its supporting result-specific rationale and independent reassessment remain unavailable. No historical score was changed. The matrix and verification qualifications are provided in Supplementary File supplementary, Section SF.

The seven-domain ROBINS-E matrix from the supplied review was retained for transparent reporting [27]. Figure 3 displays these recorded single-investigator judgements, and Supplementary File supplementary, Section SF, provides the domain-level matrix. They have not undergone independent verification. The default overall judgement follows the highest-risk domain, but a justified cumulative upward override is permitted. Thomas’s historical very-high overall rating is therefore not automatically invalid; its result-specific rationale remains unavailable. Thomas is contextual evidence and its historical rating is not used to determine certainty for an adult-only, deep-infection timing comparison. The published ROBINS-E version addresses follow-up studies; Haile’s case-control design requires a design-appropriate appraisal rather than an assumption that the historical ROBINS-E rating has been validated. No new signalling-question assessment is claimed. Reproducing the totals in the matrix is not an independent evaluation of the domain judgements or their supporting rationale.

**Table 1:** Reports with a timing-cutoff comparison and their applicability to the review question.

Study	Population	Fracture Type	Time Origin	Threshold	Outcome	Follow-Up	Effect Estimate (95% CI)
Al-Arabi 2007 [32]	133; age restriction NR	Mixed long bones	NR	360 min	Clinical infection	NR	5.7% vs. 22.2%; ratio NR
Zumsteg 2014 [33]	200 adults	Radius/ulna	Initial timing; origin NR	180 min	Deep infection/I&D	Up to 6 mo; minimum NR	6% vs. 2%; ratio NR
Lack 2015 [25]	137	GA III tibia	Injury	66 min	Deep infection	90 d	aOR 3.78 (1.16–12.31)
Hendrickson 2020 [21]	156 adults/159 fractures	GA IIIB tibia	Injury	60/180 min	Deep infection	Min. 12 mo; median 26 mo	Coefficients: 60 min 0.1906 (−0.0363 to 0.4175); 180 min −0.0105 (−0.1188 to 0.0978); not ORs
Roddy 2020 [24]	230; age restriction NR	Open extremity	Hospital/ED; facility status NR	120 min; ROC-derived	SSI	Min. 30 d; SSI endpoint within 90 d	aHR 2.4 in abstract; *p* = 0.036; original CI unverified
Zuelzer 2021 [18]	127 adults analysed; corrected cohort	GA I–II–IIIA tibia; primary closure	Injury; earliest documented dose	150 min; derived at 154 min	CDC deep SSI	Min. 6 wk	Reported OR 5.60 (1.40–22.20); model attribution NR
Grigorian 2023 [34]	3367 adults; no transfers	Femur/tibia	Receiving centre	60 min	SSI/osteomyelitis composite	Inpatient	Adjusted analysis; ratio/CI NR
Okhuereigbe 2025 [23]	191 adults	GA III tibia	Hospital/ED; facility status NR	60/180 min	Deep SSI/I&D	90 d	aOR 1.02 (0.39–2.68)/1.08 (0.35–3.37)
Kock 2025 [20]	227 patients/237 fractures; age 15–63 y	Tibia shaft; 33 gunshot fractures	Study hospital ED; prior-facility status NR	3 h; equality unresolved	Consensus FRI	Min. 3 mo after definitive care	Crude OR 1.2 (0.5–2.9); whole-cohort contextual
Haile 2025 [26]	84 cases/168 controls	Long bones	Injury	3–12 h/>12 h; ref. <3 h	Consensus FRI	Min. 6 mo	aOR 1.16 (0.35–4.13)/4.92 (1.63–16.90)
Morgan 2026 [19]	477 adults; 270 within 1 h/207 later; no transfers	Blunt long-bone	Receiving centre	60 min	Deep infection	Common window NR	Crude/derived OR 1.13 (0.66–1.93)
Sulala 2026 [22]	115 patients; age 13–70 y	Femur; SIGN nail; 82 bullet injuries	Injury	≥6 h vs. <6 h	Clinical FRI; consensus uncertain	≥1 visit; scheduled to 12 mo	Printed aOR 3.981 (1.035–15.317); contextual

### 2.10. Certainty of Evidence

GRADE was organised by timing comparison and time origin, distinguishing the primary adult deep-infection question from contextual evidence [35]. Observational evidence began at low certainty. The existing profiles state the concerns about risk of bias, indirectness, and imprecision. Inconsistency is not assessable for a single study, with no downgrade solely because only one study is available. Publication bias is not inferred from sparse groups, and selective-reporting concerns are not counted twice. Where an eligible evidence body is unestablished, no primary certainty rating is assigned. Supplementary File supplementary, Section SG, preserves the historical profiles with the later Kock and Roddy applicability qualifications. The profiles are recorded assessments with explicit limitations, not independently validated final evidence profiles. Very low certainty denotes very limited confidence in the reported associations, not evidence that earlier and later treatment are equivalent or that a longer delay is safe. Limitations of the review process are disclosed separately from the risk of bias in the primary studies. A very-low-certainty label does not compensate for an incomplete search or an extraction error. The newly recovered Hendrickson coefficient intervals supersede any earlier rationale based on their absence; they do not establish a log-odds scale or independently validate a revised GRADE judgement.

### 2.11. Synthesis Without Meta-Analysis

This synthesis uses published study-level results, not newly collected participant data or individual-participant-data reanalysis. The analytical sequence was: establish population, anatomy, mechanism, outcome, and follow-up applicability; identify the true first dose and time origin; retain the reported cutoff, boundary, and reference category; distinguish the effect measure and covariate adjustment; link multiple reports or cutoffs from the same cohort; and display interpretable comparisons with their precision and limitations. Scheme S2 in Supplementary File supplementary, Section SK, documents this analytical sequence. Individual estimates were not pooled or counted according to statistical significance. Scarcity refers to compatible information within a given clinical comparison, not a verified global count of relevant publications.

The earlier two-study 180 min pooled estimate was removed during peer-review revision. One contributing odds ratio was adjusted and the other was crude; differences in the populations, outcome ascertainment, and adjustment sets meant that the two estimates could not be interpreted as estimates of a common underlying effect. No pooled estimate, weighting diamond, or heterogeneity statistic is retained. The previously reported I^2^ of zero did not demonstrate clinical homogeneity: with only two studies, statistical heterogeneity is poorly characterised and does not resolve differences in confounding or outcome measurement [16].

Clinical comparability was assessed before any consideration of statistical pooling. Differences in time zero, age eligibility, fracture severity and site, contamination, transfers, antibiotic regimen, infection definition, follow-up, and covariate adjustment prevented a common-effect interpretation. The available records were insufficient for formal interaction tests, risk-of-bias-restricted meta-analysis, or long-term pooled analysis. Restricted populations are described narratively without implying that a subgroup effect was established.

Figure 2 presents individual odds ratios without a summary effect. Kock and Sulala remain as whole-cohort contextual evidence, not verified adult-only, nonballistic comparisons. Sulala is plotted from the exact printed Table 3 of [22] values, 3.981 (95% CI 1.035–15.317), with labels rounded to two decimals. Hendrickson’s coefficients and confidence intervals are retained on their original scale in Table 1 of [21] and are not converted to odds ratios. Roddy’s abstract-reported hazard ratio is not assigned an unverified original-model confidence interval. Source-transcription checks and calculations from aggregate cells are documented separately from the uncompleted independent human assessment. No new pooled analysis was performed.

### 2.12. Reporting Bias

No funnel plot or small-study-effect test was performed. The available groups were too sparse, and several did not share a common estimand. Missing database exports also prevent a reliable assessment of search completeness. Data-derived cutoffs and incompletely documented analyses are therefore identified as potential reporting-bias concerns, without claiming that selective publication has been demonstrated.

## 3. Results

### 3.1. Study Selection

The preserved inventories contain 24 entries (11 + 13). The earlier audit set aside three reviews and three conference/companion entries, recorded 18 candidate reports, and retained 16 reports after two recorded exclusions. These numbers describe historical classifications, not complete database yields or independently verified inclusion decisions. The 16 named reports remain identified as I01–I16 in Supplementary File supplementary, Section SC. Table 1 contains 12 timing-comparison reports; Dellinger [28], Patzakis [29], Thomas [30], and Johnson [31] are documented separately as contextual reports. No report has been added merely to reconcile a total. Historical dispositions, including the report by Leonidou et al. [36], and unresolved companion relationships are reported with their evidential limitations.

The inventory arithmetic does not establish that all 18 full reports were retrieved or that 16 studies satisfied the target eligibility criteria. Figure 1 retains the supplied PRISMA-style layout but identifies the numbers as prior-inventory counts. Unverified zero-yield and retrieval labels, and the unsupported statement that 13 studies provided adult-only data, have been removed. A separate source-verification queue described on 22 September 2026 contains 36 entries, including the original 24 and 12 additional source-linked candidates; it includes reviews, contextual reports, and unresolved candidates. It is not a search yield or an included-study total, and it is not incorporated into the historical figure. A complete search-to-inclusion flow has not been established.

### 3.2. Characteristics of Reports in the Evidence Inventory

The historical evidence map contains 16 reports published from 1988 to 2026. Open-tibia cohorts predominate, alongside forearm, femoral, mixed-fracture, and process-improvement reports. Table 1 summarises timing-cutoff reports; the extended contextual inventory is retained in the supplements. Kock’s full-text methods exclude skeletal immaturity, rather than explicitly requiring age of at least 18 years. Its results report 227 patients with 237 fractures, an age range of 15–63 years, and 33 gunshot fractures [20]. No age-by-timing-by-FRI table or eligible adult, nonballistic subgroup was identified in the accessed full text. These limitations prevent treating its whole-cohort estimate as directly applicable to the primary question. Sulala likewise cannot be treated as an adult-only, nonballistic cohort: its results report ages 13–70 years and 82 bullet injuries among 115 patients [22]. No separable target subgroup was identified in the accessed report.

The studies differed in direct-admission and transfer pathways, fracture severity, contamination, antibiotic regimens, infection definitions, surveillance periods, and adjustment sets. These differences are shown rather than reduced to a single heterogeneity statistic. The number of retained reports should not be read as the number of comparable adult-only effect estimates.

The 12 reports are not 12 confirmed eligible adult-only studies. Kock and Sulala remain as mixed-age, mixed-mechanism contextual evidence. Hendrickson’s coefficient intervals are transcribed on their original scale; they are not ORs. Sulala’s exact printed Table 3 of [21] result and ≥6 h boundary supersede the earlier rounded extraction; a conflicting crude-OR row remains unresolved. Roddy’s original-model confidence limits remain unverified. Source-specific details and the four further contextual reports appear in Supplementary File supplementary.

### 3.3. Time-Origin Classification and Transfer-Related Exposure Misclassification

Injury-based intervals include prehospital delay. The receiving-centre analyses by Grigorian and Morgan excluded transfers, whereas several other records described ED or hospital arrival without resolving the position of that facility within the transfer pathway. These arrival-based records were not reclassified as injury-to-antibiotic intervals. No separate first-medical-contact threshold estimate was identified in the preserved effect inventory.

Legacy reports with unclear time zero remained unresolved. The targeted Zuelzer full-text check identified an injury-to-first-dose interval using ambulance, transfer, and hospital records; the earliest documented dose was used [18]. This replaces its earlier ambiguous injury/admission label. Supplementary Figure S1 illustrates why a first dose given before transfer cannot be replaced by a later receiving-centre dose when defining the exposure.

### 3.4. Outcome Definitions and Follow-Up

Kock and Haile describe consensus FRI, while other reports use deep infection requiring debridement, CDC/NHSN SSI, inpatient composites, or clinical infection without a common definition. Sulala defined infection from clinical documentation and acknowledged that ascertainment might not fully align with standardised FRI criteria [22]; it is not classified here as confirmed strict-consensus FRI. Kock requires at least three months of follow-up after definitive management [20]. Thomas reports an infection-or-nonunion outcome rather than an isolated deep-infection endpoint and includes patients aged 1–89 years [30]. Mixed populations and composite outcomes are distinguished from the primary adult deep-infection question; limited applicability does not, by itself, establish biased measurement within the original study.

Surveillance varied from a minimum of 21 days to a minimum of 12 months. Hendrickson provided the clearest minimum 12-month follow-up, but its reported endpoint was deep infection rather than a confirmed consensus–FRI definition. Kock and Haile had shorter minimum follow-up, and Sulala described scheduled surveillance to 12 months without confirming complete minimum 12-month ascertainment. Thus, the available extraction does not establish a body of three studies meeting both strict-consensus FRI and a minimum 12-month follow-up. Roddy’s abstract specifies a minimum follow-up of 30 days and an SSI endpoint within 90 days; these are distinct quantities. The earlier 90-day-only entry was corrected rather than interpreted as complete minimum-90-day surveillance [24].

### 3.5. Causal Adjustment and Confounding by Indication

Multivariable timing analyses were reported in Lack, Roddy, Grigorian, Okhuereigbe, Haile, and Sulala, but covariates and outcome windows differed. Okhuereigbe included Injury Severity Score, Gustilo–Anderson grade, and contamination; these address measured injury-related confounding, not all host or system effects. The model corresponding to Zuelzer’s displayed 150 min OR remains unconfirmed and is not relabelled as adjusted [18]. Regimen variation is also relevant: Morgan describes cefazolin for type I–II injuries, cefazolin plus gentamicin for type III before a protocol change, and ceftriaxone after the change, with vancomycin added for highly contaminated injuries [19]. These are study-reported practices, not a regimen recommendation from this review, and timing comparisons across protocol periods may also reflect treatment differences.

The Grigorian extraction describes a higher crude infection frequency in the early-antibiotic group and attenuation after covariate adjustment [34]. This pattern is compatible with confounding by indication, but attenuation alone neither proves adequate adjustment nor establishes the causal effect of treatment delay.

### 3.6. Threshold-Specific Associations

#### 3.6.1. Exact 60 Min Threshold

The 60 min evidence comprised injury-based regression results from Hendrickson, an adjusted ED-arrival comparison from Okhuereigbe, and receiving-centre reports from Grigorian and Morgan [19,21,23,34]. The Okhuereigbe estimate was imprecise and compatible with materially different associations. Grigorian reported an adjusted analysis of an inpatient composite rather than a harmonised longer-term deep-infection endpoint. The extracted measures and surveillance windows were not interchangeable.

Morgan’s two mutually exclusive groups account for all 477 adults: 270 received antibiotics within one hour of receiving-centre arrival and 207 after one hour (270 + 207 = 477) [19]. Deep infection occurred in 34 and 29 patients, respectively, leaving 236 and 178 without deep infection. These denominators agree with Table 1; no patient or event count was changed. The delayed-versus-earlier crude OR is displayed in Table 1 and Figure 2, with the cell-based calculation in Supplementary File supplementary, Section SK. It is not adjusted. Hendrickson’s regression coefficient is reported separately and is not interchangeable with an odds ratio. A later source check documented that the printed early-treatment anatomical subgroup events sum to 38 rather than the overall 34. The anatomical subgroup cells were not used to derive new effects or silently corrected. The overall 34/270 and 29/207 counts agree between the source text and Table 2 of Morgan et al. [19]; the discrepancy is documented in Supplementary File supplementary, Section SE.5.

#### 3.6.2. Exact 120 Min Threshold

Roddy supplied the only extracted 120 min comparison [24]. The primary abstract reports an adjusted Cox HR of 2.4 (*p* = 0.036), with adjustment for age, smoking, and drug use, for administration after 120 min from ED presentation. The cutoff was ROC-derived. Minimum follow-up was 30 days, whereas the SSI endpoint was defined within 90 days. The original model’s exact 95% CI was not verified. The approximate interval previously displayed in the manuscript has been removed rather than treated as an exact published estimate. Secondary citation of an interval and the differing publisher Discussion description are documented in Supplementary File supplementary, Section SE.1. Roddy is retained as qualified narrative evidence without a CI bar, standard-error reconstruction, or pooling weight.

#### 3.6.3. Exact 180 Min Threshold

The three reports highlighted for 180 min address different comparisons [20,21,23]. Hendrickson uses time from injury and reports a non-ratio regression coefficient; Okhuereigbe reports an adjusted hospital/ED-arrival OR; Kock reports a crude hospital-arrival OR for its unseparated mixed-age, mixed-mechanism fracture cohort. Therefore, three reports mentioning the same numerical cutoff do not provide three interchangeable ORs. The former pool used only the two arrival-based ORs, but that pool has now been withdrawn because it did not support a common interpretable estimand. The current manuscript presents study-specific results only. Kock’s adult/nonballistic subgroup, equality at three hours, prior-facility dosing, and bilateral-fracture handling remain unresolved. Table 2 of Hendrickson et al. [21] reports coefficients of 0.1906 (95% CI −0.0363 to 0.4175) within one hour and −0.0105 (95% CI −0.1188 to 0.0978) within three hours. These intervals are presented as printed; unresolved model scale and reference coding preclude treating the coefficients as log odds ratios.

Other historical reports, including Dellinger, Patzakis, and Zumsteg, also mention three hours, but their population, time-origin, outcome, or surveillance limitations prevent treating these as one comparable group. Haile’s three-to-12 h contrast retains the below-three-hour reference and is not collapsed into a new binary 180 min effect. Supplementary File supplementary distinguishes the numerical cutoff from clinical comparability.

#### 3.6.4. Other/Data-Derived and Long-Delay Comparisons

Study-specific associations were observed at several non-equivalent thresholds: 66 min in Lack, 150 min in Zuelzer, six hours in Sulala, and the categories below three, three to 12, and above 12 h in Haile [18,22,25,26]. The primary-study populations, reference categories, outcome windows, and models differed. The three-to-12 h Haile comparison is retained alongside the above-12 h comparison to avoid selecting only the more pronounced association. These cutoffs do not identify a common biological threshold. Zuelzer’s linked erratum clarifies an initial 142 patients with 143 fractures, followed by exclusion of 15 patients with 16 fractures for insufficient follow-up, leaving 127 patients for analysis [18]. The erratum corrects patient-flow reporting; it does not describe a retraction, supply a replacement timing model, or contribute an additional cohort. Its reported 150 min OR is retained as a published association with unresolved model attribution, not as a validated causal cutoff. Its six-week minimum follow-up and unresolved model attribution limit its applicability. Sulala’s six-hour comparison remains as whole-cohort contextual evidence, not an eligible adult, nonballistic estimate [22]. Table 3 of Sulala et al. [22] prints an aOR of 3.981 (95% CI 1.035–15.317; *p* = 0.044) for ≥6 versus <6 h. The conflicting crude-OR row is reported separately rather than reconciled by changing a source value.

Continuous-time analyses were described by Hendrickson and Okhuereigbe [21,23]. Table 2 of Hendrickson et al. [21] gives an injury-to-antibiotic coefficient of −0.0003 (95% CI −0.0010 to 0.0004). The coefficient is retained on its printed scale, without conversion to an hourly OR. Differences in units, model specification, and time origin prevent a common interpretation. These recovered intervals do not resolve the model ambiguity or independently validate the extraction.

### 3.7. Restricted and Contextual Evidence

Severe open-tibia cohorts examined different cutoffs using injury-based or hospital-arrival intervals. Direct-admission cohorts and transfer-dependent settings also differed in case mix and ascertainment. These restricted populations do not constitute formal interaction analyses, and differences in statistical significance between reports were not interpreted as evidence of effect modification.

The available inventory did not support a verified lower-risk, adjusted-only, long-term, or strict-FRI pooled analysis. No leave-one-out analysis was retained after withdrawal of the pooled estimate. The reasons for not combining each timing group are documented in the supplementary applicability and GRADE tables.

### 3.8. Risk of Bias and Certainty of Evidence

The recorded overall ROBINS-E categories sum up to 16 reports: one with some concerns, 12 with high risk, and three with very high risk. Confounding was high or very high in 15. A row-by-row counting check reproduced these totals; it did not validate the underlying appraisals. The matrix is now read in the explicit order study, D1–D7, the overall in Supplementary File supplementary, Section SF, and displayed in Figure 3. Thomas’s very-high overall judgement exceeds its highest individual-domain category, which can be permissible when several high-risk problems are cumulatively severe [27]. Its original result-specific rationale remains unavailable. The historical score has neither been retrospectively justified by counting domains nor silently changed. Thomas remains contextual and does not determine certainty for an adult-only deep-infection comparison.

Historical GRADE profiles classified assessable timing comparisons as very low certainty. No combined rating is assigned to non-equivalent long-delay contrasts or an unestablished minimum-12-month consensus-FRI evidence body. Neither Kock’s nor Sulala’s unseparated mixed-age, mixed-mechanism comparison receives an adult-only primary certainty rating. Roddy remains indirect for the minimum-90-day deep-infection question, with original-model precision unresolved. Thomas’s population and composite endpoint concern applicability and do not themselves validate its ROBINS-E ratings. Supplementary File supplementary, Section SG, presents the qualified profiles; the independent confirmation remains outstanding.

## 4. Discussion

### 4.1. Principal Findings

The reported timing associations are difficult to interpret because of confounding, inconsistent exposure definitions, and heterogeneous populations and outcomes. Recorded ROBINS-E and GRADE assessments indicate substantial concerns but have not been independently verified. Consequently, neither covariate adjustment nor an apparently similar direction of results establishes a causal threshold. The withdrawn 180 min pooled estimate remains excluded. The contribution of the current synthesis is to show which reported comparisons address different clinical questions and why they should not be treated as interchangeable. This interpretation is confined to the preserved evidence and does not establish the completeness of the literature.

### 4.2. A 60 Min Quality Metric Is Not a Biological Threshold

An operational quality metric specifies a measurable target within a care pathway. A biological threshold would imply a change in infection risk attributable to crossing a particular elapsed time, under comparable injury and treatment conditions. These are different propositions. The present observational comparisons do not identify the shape of the causal time–risk relationship and cannot establish either the existence or the absence of a discontinuity at 60 min.

A 60 min target can still be used to organise prompt care without being interpreted as a proven biological cutoff. Its definition must specify the starting point and the true first dose. Failure to meet a target should prompt examination of the care pathway, not an assumption that every patient crossing the boundary has experienced the same biological increase in risk. Likewise, an imprecise association does not show that a longer delay is safe.

### 4.3. Confounding by Indication and Causal Interpretation

Confounding by indication is central to interpretation. Severe contamination, vascular injury, and major trauma may prompt earlier antibiotics while also increasing infection risk. Conversely, transfer delays, competing resuscitation priorities, and limited resources can prolong time to treatment and independently affect outcomes. Adjustment can address measured differences, but incomplete covariates, measurement error, and selection remain.

The causal framework is an interpretive aid, not evidence that these pathways were adequately controlled in the contributing studies. Post-exposure surgery and soft-tissue management also require an explicit causal rationale before adjustment. The revised synthesis therefore reports model characteristics and does not use the word “adjusted” as a synonym for “causal”.

### 4.4. Outcome Definition and Surveillance Duration

Deep infection, consensus FRI, superficial wound infection, and composites that include nonunion are not interchangeable. Combining them would change the clinical question. Similarly, minimum follow-up, median follow-up, scheduled visits, and inpatient surveillance describe different opportunities to detect infection and should not be treated as equivalent.

The supplied extraction confirms a minimum 12-month deep-infection follow-up in Hendrickson but does not establish a set meeting both strict-consensus FRI and that minimum duration. The earlier three-study long-term strict-FRI label has therefore been withdrawn. Longer, consistently ascertained follow-up remains important because late infections may be missed by 90-day surveillance [5].

### 4.5. Clinical and Trauma-System Implications

These findings do not support delaying systemic antibiotics. Prompt prophylaxis remains consistent with open-fracture guidance [7,8,9]. Process reporting can distinguish injury-to-dose and facility-arrival-to-dose intervals, document pretransfer doses, and describe delays by injury severity and transfer pathway. These are proposals for clearer measurement, not interventions whose benefit was tested by this review.

In transfer-dependent systems, the first treating team may administer antibiotics well before arrival at the definitive trauma centre. A later dose must not be counted as the first simply to start a new clock. Agent, dose, and indication should accompany the timing data so that rapid administration is not confused with confirmed regimen appropriateness.

### 4.6. Relation to Previous Reviews

Whitehouse et al. synthesised eight timing studies, Chan et al. found no randomised comparison of initial-dose timing within a broader Cochrane review of open-fracture care, and Marchiori and Nunes retained 14 heterogeneous studies without pooling [10,11,12]. The present synthesis makes the study-specific time origins, cutoffs, adjustment, age applicability, and outcome windows explicit. Its contribution is interpretability of those comparisons, not proof of a new biological threshold or a verified exhaustive update. The missing search exports and absence of independent review remain substantive limitations.

Operative-timing and treatment-audit reports require assessment beyond their titles [37,38,39]. Enninghorst prospectively recorded antibiotic timing and deep infection, and Weber’s abstract reports an adjusted antibiotic-timing OR; both require full-report eligibility and model review. Table 2 of Assunção et al. [39] reports infectious complications in 27/172 patients treated within three hours of admission and 18/69 treated later [39]. These are not verified isolated deep-infection outcomes in the target long-bone, nonballistic, minimum-90-day population. Their presence strengthens the need for renewed selection rather than establishing a new included-study count. Supplementary File supplementary, Section SE.8 records these candidates and the source-limited first-pass assessments.

### 4.7. Strengths and Limitations

The principal analytic contribution is the explicit separation of time origins, cutoff boundaries, effect measures, population applicability, infection definitions, and surveillance periods. This makes the limits of individual comparisons visible and avoids producing a pooled result with no coherent clinical interpretation. It is a contribution to interpretation of the preserved reports, not evidence that the search or review process met all systematic-review standards.

Two review-level limitations remain unresolved. First, complete source-specific exports and a reproducible database-search-to-inclusion trail are unavailable; the 24-entry historical inventory cannot establish comprehensive coverage. Omitted studies could alter the apparent direction, consistency, or clinical applicability of the findings. Second, eligibility, time-origin classification, numerical extraction, ROBINS-E, and GRADE were not independently verified. Same-investigator and AI-assisted checks cannot exclude repeated classification or transcription errors. These limitations remain relevant without meta-analysis and are not neutralised by a very-low-certainty rating. The retrospective protocol also cannot establish that analytic decisions preceded knowledge of results. Kock and Sulala lack separable target-population data; Roddy’s original-model precision and Thomas’s cumulative appraisal rationale remain unresolved. The recovered Hendrickson intervals and other source corrections improve selected entries but do not validate the evidence set as a whole. The present report should therefore be interpreted as a limited reconstruction with study-specific evidence synthesis, not an exhaustive or independently verified account.

### 4.8. Research Priorities

Before a definitive update, the database strategies should be executed with preserved native exports and independently verified selection, extraction, and appraisal. Future primary studies should record injury, first-contact, each facility-arrival, and every antibiotic-dose time, together with the regimen, transfer status, fracture severity, contamination, host factors, and subsequent care. Consensus FRI with complete minimum 12-month surveillance would address an important evidence gap.

Future analyses should define the estimand and covariate strategy prospectively, model time continuously where appropriate, and evaluate externally specified contrasts without selecting cutoffs according to the favourable results. A harmonised multicentre cohort or individual-participant-data collaboration may support this approach. Implementation studies can evaluate faster delivery pathways without deliberately withholding indicated antibiotics.

## 5. Conclusions

The preserved reports show why a 60 min operational quality target should not be equated with a validated biological infection threshold. Their observational associations establish neither causal cutoffs at 60, 120, or 180 min nor equivalence or safe delay. Indicated systemic prophylaxis should not be postponed on the basis of these findings. Incomplete searches, non-independent assessment, confounding, and heterogeneous outcome ascertainment substantially limit the completeness and reliability of this evidence synthesis.

## Data Availability

The protocol was retrospectively archived on OSF (DOI: 10.17605/OSF.IO/7J8QN) [17], with associated project https://osf.io/g3vma (accessed on 30 September 2026) and preservation copy https://archive.org/details/osf-registrations-7j8qn-v1 (accessed on 30 September 2026). The reported registration licence is CC0 1.0 Universal. The accompanying local revision package contains the preserved bibliographic inventories, prepared search strategies, source-derived summaries, recorded appraisals, figures, and a change crosswalk. Complete native search exports, locked paired independent assessments, and author-supplied subgroup data are unavailable. Upload of the present revision to the associated OSF project has not been performed or verified. Copyrighted primary-study PDFs and private correspondence are not redistributed.

## Supplementary Materials

The following supporting information can be downloaded at: https://www.mdpi.com/article/10.3390/jcm15197689/s1, Supplementary File supplementary: Consolidated provenance, source corrections, and appraisal qualifications; Table S1: Chronology and version precedence; Table S2: Planned information sources and recorded search status; Table S3: Historical report inventory and analytic qualifications; Table S4: Documented checks and independent-review status; Table S5: Recorded ROBINS-E judgements; Figure S1: Distinct time origins and the true first-dose endpoint. Retained from the earlier revision; no additional clinical result is implied; Table S6: Round 2 changes and reporting map; Table S7: Aggregate arithmetic checks and their limitations; PRISMA 2020 Checklist: Completed PRISMA 2020 reporting checklist [13]. Supplementary File supplementary provides the consolidated record of the chronology, source status, historical report identifiers, audit scope, source-specific corrections, recorded ROBINS-E assessments, qualified GRADE profiles, and analytical framework. The supplied materials document the basis and limitations of this revision; they do not constitute complete native database exports or completed paired independent-review records.

## References

[1] Court-Brown C.M., Bugler K.E., Clement N.D., Duckworth A.D., McQueen M.M. (2012). The epidemiology of open fractures in adults. A 15-year review. Injury.

[2] Gustilo R.B., Anderson J.T. (1976). Prevention of infection in the treatment of one thousand and twenty-five open fractures of long bones. J. Bone Jt. Surg. Am..

[3] Metsemakers W.J., Morgenstern M., McNally M.A., Moriarty T.F., McFadyen I., Scarborough M., Athanasou N., Ochsner P., Kuehl R., Raschke M. (2018). Fracture-related infection: A consensus on definition from an international expert group. Injury.

[4] Govaert G.A.M., Kuehl R., Atkins B.L., Trampuz A., Morgenstern M., Obremskey W.T., Verhofstad M.H.J., McNally M.A., Metsemakers W.-J. (2020). on behalf of the Fracture-Related Infection (FRI) Consensus Group. Diagnosing fracture-related infection: Current concepts and recommendations. J. Orthop. Trauma.

[5] Zalavras C.G., Aerden L., Declercq P., Belmans A., Metsemakers W.J. (2022). Ninety-day follow-up is inadequate for diagnosis of fracture-related infections in patients with open fractures. Clin. Orthop. Relat. Res..

[6] Pley C., Purohit K., Krkovic M., Abdulkarim A. (2020). A health economic analysis of the management of open lower limb fractures in the elderly. Eur. J. Orthop. Surg. Traumatol..

[7] Gosselin R.A., Roberts I., Gillespie W.J. (2004). Antibiotics for preventing infection in open limb fractures. Cochrane Database Syst. Rev..

[8] American Academy of Orthopaedic Surgeons (2022). Prevention of Surgical Site Infections After Major Extremity Trauma: Evidence-Based Clinical Practice Guideline.

[9] British Orthopaedic Association Trauma Committee (2020). British Orthopaedic Association Standard for Trauma (BOAST): Open fracture management. Injury.

[10] Whitehouse M.R., McDaid C., Kelly M.B., Moran C.G., Costa M.L. (2017). The effect of timing of antibiotic delivery on infection rates related to open limb fractures: A systematic review. Emerg. Med. J..

[11] Chan J.K.-K., Aquilina A.L., Lewis S.R., Rodrigues J.N., Griffin X.L., Nanchahal J. (2022). Timing of antibiotic administration, wound debridement, and the stages of reconstructive surgery for open long bone fractures of the upper and lower limbs. Cochrane Database Syst. Rev..

[12] Marchiori J.G.T., Nunes A.P.F. (2024). Time until the start of antibiotic prophylaxis and the risk of open fracture infection: A systematic review. Acta Ortop. Bras..

[13] Page M.J., McKenzie J.E., Bossuyt P.M., Boutron I., Hoffmann T.C., Mulrow C.D., Shamseer L., Tetzlaff J.M., Akl A.E., Brennan S.E. (2021). The PRISMA 2020 statement: An updated guideline for reporting systematic reviews. BMJ.

[14] Rethlefsen M.L., Kirtley S., Waffenschmidt S., Ayala A.P., Moher D., Page M.J., Koffel J.B. (2021). PRISMA-S: An extension to the PRISMA Statement for Reporting Literature Searches in Systematic Reviews. Syst. Rev..

[15] Stroup D.F., Berlin J.A., Morton S.C., Olkin I., Williamson G.D., Rennie D., Moher D., Becker B.J., Sipe T.A., Thacker S.B. (2000). Meta-analysis of observational studies in epidemiology: A proposal for reporting. JAMA.

[16] Higgins J.P.T., Thomas J., Chandler J., Cumpston M., Li T., Page M.J., Welch V.A. (2024). Cochrane Handbook for Systematic Reviews of Interventions.

[17] Nguyen T.V. (2026). Retrospective Protocol Archive for Timing of First Systemic Antibiotic Administration and Risk of Deep Infection in Adults with Open Long-Bone Fractures: A Systematic Review and Threshold-Specific Meta-Analysis. Preprints.

[18] Zuelzer D.A., Hayes C.B., Hautala G.S., Akbar A., Mayer R.R., Jacobs C.A., Wright R.D., Moghadamian E.S., Matuszewski P.E. (2021). Early antibiotic administration is associated with a reduced infection risk when combined with primary wound closure in patients with open tibia fractures. Clin. Orthop. Relat. Res..

[19] Morgan D., Orie G., Jones C., Lutnick E., Nader N.D., Anders M. (2026). Early prophylactic antibiotics effect on infection rate in blunt open fractures. J. Orthop. Surg. Res..

[20] Kock F.W., Basson T.R., Burger M.C., Ferreira N. (2025). The effect of treatment delays on fracture-related infection in open tibia shaft fractures: A retrospective cohort study. Strateg. Trauma Limb Reconstr..

[21] Hendrickson S.A., Donovan R., Harries L., Wright T.C., Whitehouse M.R., Khan U. (2020). Time to intravenous antibiotic administration (TIbiA) in severe open tibial fractures: Impact of change to national guidance. Injury.

[22] Sulala A.C., Tsegaye Y.A., Kefale W.M., Tegegne B.B., Wubie A.G., Taffere S.N., Zegergsh A.G., Awedew A.F., Tsegaye G.W. (2026). Incidence and risk factors of fracture-related infection after SIGN nailing of open femoral fractures in Ethiopia. Sci. Rep..

[23] Okhuereigbe D., Raffetto M., Li V., Bell A., Lawrence J.E., Kim J., Stennett C.A., Slobogean G.P., O’Hara N.N.P., O’Toole R.V. (2025). Association between timing of antibiotics and deep surgical site infection in Gustilo-Anderson type III open tibia fractures. J. Orthop. Trauma.

[24] Roddy E., Patterson J.T., Kandemir U. (2020). Delay of antibiotic administration greater than 2 hours predicts surgical site infection in open fractures. Injury.

[25] Lack W.D., Karunakar M.A., Angerame M.R., Seymour R.B., Sims S., Kellam J.F., Bosse M.J. (2015). Type III open tibia fractures: Immediate antibiotic prophylaxis minimizes infection. J. Orthop. Trauma.

[26] Haile A.M., Dagnaw B., Gebeyehu M., Mengesha S., Yoseph T., Nigussie H., Woldemariam T., Wamisho B.L., Zeki K. (2025). Fracture-related infection in open long-bone fractures: A bicenter case-control study in a limited-resource country. JBJS Open Access.

[27] Higgins J.P.T., Morgan R.L., Rooney A.A., Taylor K.W., Thayer K.A., Silva R.A., Lemeris C., Akl E.A., Bateson T.F., Berkman N.D. (2024). A tool to assess risk of bias in non-randomized follow-up studies of exposure effects (ROBINS-E). Environ. Int..

[28] Dellinger E.P., Miller S.D., Wertz M.J., Grypma M., Droppert B., Anderson P.A. (1988). Risk of infection after open fracture of the arm or leg. Arch. Surg..

[29] Patzakis M.J., Wilkins J. (1989). Factors influencing infection rate in open fracture wounds. Clin. Orthop. Relat. Res..

[30] Thomas S.H., Arthur A.O., Howard Z., Shear M.L., Kadzielski J.L., Vrahas M.S. (2013). Helicopter emergency medical services crew administration of antibiotics for open fractures. Air Med. J..

[31] Johnson J.P., Goodman A.D., Haag A.M., Hayda R.A. (2017). Decreased time to antibiotic prophylaxis for open fractures at a Level One Trauma Center. J. Orthop. Trauma.

[32] Al-Arabi Y.B., Nader M., Hamidian-Jahromi A.R., Woods D.A. (2007). The effect of the timing of antibiotics and surgical treatment on infection rates in open long-bone fractures: A 9-year prospective study from a district general hospital. Injury.

[33] Zumsteg J.W., Molina C.S., Lee D.H., Pappas N.D. (2014). Factors influencing infection rates after open fractures of the radius and/or ulna. J. Hand Surg. Am..

[34] Grigorian A., Schellenberg M., Inaba K., Martin M., Matsushima K., Lekawa M., Nahmias J.M. (2023). Antibiotic administration within 1 hour for open lower extremity fractures is not associated with decreased risk of infection. J. Trauma Acute Care Surg..

[35] Balshem H., Helfand M., Schunemann H.J., Oxman A.D., Kunz R., Brozek J., Vist E.G., Falck-Ytter Y., Meerpohl J., Norris S. (2011). GRADE guidelines: 3. Rating the quality of evidence. J. Clin. Epidemiol..

[36] Leonidou A., Kiraly Z., Gality H., Apperley S., Vanstone S., Woods D.A. (2014). The effect of the timing of antibiotics and surgical treatment on infection rates in open long-bone fractures: A 6-year prospective study after a change in policy. Strateg. Trauma Limb Reconstr..

[37] Enninghorst N., McDougall D., Hunt J.J., Balogh Z.J. (2011). Open tibia fractures: Timely debridement leaves injury severity as the only determinant of poor outcome. J. Trauma.

[38] Weber D., Dulai S.K., Bergman J., Buckley R., Beaupre L.A. (2014). Time to initial operative treatment following open fracture does not impact development of deep infection: A prospective cohort study of 736 subjects. J. Orthop. Trauma.

[39] Assunção A.L.F., Oliveira de S.T. (2020). Clinical audit of primary treatment of open fractures: Antibiotic treatment and tetanus prophylaxis. Rev. Bras. Ortop..

