# Supplementary material for: Timing of First Systemic Antibiotic Administration and Risk of Deep Infection in Adults with Open Long-Bone Fractures: A Systematic Review and Threshold-Specific Evidence Synthesis
Source: J Clin Med. 2026 Oct 4;15(19):7689. doi: 10.3390/jcm15197689 (PMC13665906; doi:10.3390/jcm15197689)
Supplement: Supplementary file 1 [file jcm-15-07689-s001.zip › jcm-4524900 - PRISMA 2020.pdf]

PRISMA 2020 Checklist

Timing of First Systemic Antibiotic Administration and Risk of Deep Infection in Adults with Open Long-Bone Fractures: A Systematic Review and Threshold-Specific Evidence Synthesis

Nguyen The Diep; Nguyen Minh Chau; Phan Thanh Nam; Tien Van Nguyen

Section / topic	Item	Checklist item	Location and reporting note
**TITLE**			
Title	1	Identify the report as a systematic review.	**Title page, p. 1.**<br>Reported. The current title identifies a systematic review and threshold-specific evidence synthesis; no retained meta-analysis is claimed.
**ABSTRACT**			
Abstract	2	See the PRISMA 2020 for Abstracts checklist.	**Abstract, p. 1; separate PRISMA 2020 for Abstracts checklist.**<br>Partly reported. The accompanying 12-item checklist identifies the information present and the remaining abstract-level omissions.
**INTRODUCTION**			
Rationale	3	Describe the rationale for the review in the context of existing knowledge.	**Introduction, pp. 2–3; Section 4.6, p. 23.**<br>Reported. Earlier reviews, clinical relevance, non-equivalent time origins, and outcome and confounding differences are discussed.
Objectives	4	Provide an explicit statement of the objective(s) or question(s) the review addresses.	**Introduction, final paragraph, p. 3; Abstract, p. 1.**<br>Reported. The objective concerns study-specific first-dose timing associations and interpretation by time origin, cutoff and outcome.

Section / topic	Item	Checklist item	Location and reporting note
**METHODS**			
Eligibility criteria	5	Specify the inclusion and exclusion criteria for the review and how studies were grouped for the syntheses.	**Sections 2.2, 2.6 and 2.8, pp. 4–5 and 7–8; SC-R2, Sections C and K.** Criteria and grouping rules reported. Adults ≥18 years, fracture sites, systemic first-dose comparisons, outcome hierarchy and follow-up are specified. Final application to the complete literature is not verified.
Information sources	6	Specify all databases, registers, websites, organisations, reference lists and other sources searched or consulted to identify studies. Specify the date when each source was last searched or consulted.	**Section 2.3, pp. 5–6; SC-R2, Sections A–B, pp. 1–2.** Partly reported. Planned sources, preserved inventories and targeted checks are distinguished. Complete successful source-specific search dates and yields are unavailable; attempt dates are not final-search dates.
Search strategy	7	Present the full search strategies for all databases, registers and websites, including any filters and limits used.	**Section 2.3, pp. 5–6; SC-R2, Section B, pp. 1–2; accompanying search-strategy drafts.** Partly reported. Prepared strings are identified, but complete executed searches and applied interface limits cannot be confirmed. Draft strategies are not represented as successful database searches.
Selection process	8	Specify the methods used to decide whether a study met the inclusion criteria of the review, including how many reviewers screened each record and each report retrieved, whether they worked independently, and if applicable, details of automation tools used in the process.	**Section 2.4, p. 6; Acknowledgments, pp. 25–26; SC-R2, Sections C–D, pp. 2–4.** Reported with a conduct limitation. T.V.N. performed original selection; repeat checks were not independent. No paired decisions or inter-reviewer agreement are claimed. AI assistance is not a second human reviewer.

Section / topic	Item	Checklist item	Location and reporting note
Data collection process	9	Specify the methods used to collect data from reports, including how many reviewers collected data from each report, whether they worked independently, any processes for obtaining or confirming data from study investigators, and if applicable, details of automation tools used in the process.	**Sections 2.4–2.5, pp. 6–7; Abstract, p. 1; Acknowledgments, pp. 25–26; SC-R2, Sections D–E.**<br>Partly reported. The single-investigator process and source-limited checks are described. No author clarification was incorporated. Completed independent extraction and a detailed author-contact process are not documented.
Data items	10a	List and define all outcomes for which data were sought. Specify whether all results that were compatible with each outcome domain in each study were sought (e.g. for all measures, time points, analyses), and if not, the methods used to decide which results to collect.	**Sections 2.2, 2.5 and 2.8, pp. 4–8; SC-R2, Sections E and G.**<br>Partly reported. Deep infection/FRI, outcome hierarchy and surveillance rules are defined. Exhaustive collection of all eligible measures, time points and analyses within every study is not established.
Data items	10b	List and define all other variables for which data were sought (e.g. participant and intervention characteristics, funding sources). Describe any assumptions made about any missing or unclear information.	**Sections 2.2 and 2.5–2.7, pp. 4–8; Table 1, pp. 13–15; SC-R2, Sections C, E and K.**<br>Reported for the listed clinical and analytic variables. Patients/fractures, prior doses, timing, covariates and overlap are distinguished; missing values and unreported subgroups are not imputed.
Study risk of bias assessment	11	Specify the methods used to assess risk of bias in the included studies, including details of the tool(s) used, how many reviewers assessed each study and whether they worked independently, and if applicable, details of automation tools used in the process.	**Sections 2.4 and 2.9, pp. 6 and 9; SC-R2, Sections D–F, pp. 3–6; Acknowledgments.**<br>Reported with qualifications. The historical single-investigator ROBINS-E matrix is retained. Independent signalling-question assessment is absent; Thomas's overall rationale and applicability to Haile's design remain unresolved.

Section / topic	Item	Checklist item	Location and reporting note
Effect measures	12	Specify for each outcome the effect measure(s) (e.g. risk ratio, mean difference) used in the synthesis or presentation of results.	**Sections 2.5 and 2.11, pp. 6–7 and 9–10; Table 1, pp. 13–15; Figure 2, p. 19.**<br>Reported. Adjusted and crude ORs, the abstract-reported HR and original-scale coefficients are distinguished; incompatible measures are not pooled.
Synthesis methods	13a	Describe the processes used to decide which studies were eligible for each synthesis (e.g. tabulating the study intervention characteristics and comparing against the planned groups for each synthesis (item #5)).	**Sections 2.2, 2.6, 2.8 and 2.11; Table 1; SC-R2, Sections C and K.**<br>Reported grouping process. Applicability, time origin, cutoff, outcome, follow-up and adjustment guide presentation. The historical inventory is not a final independently verified eligible set.
Synthesis methods	13b	Describe any methods required to prepare the data for presentation or synthesis, such as handling of missing summary statistics, or data conversions.	**Sections 2.5–2.6 and 2.11, pp. 6–10; SC-R2, Sections E and K, pp. 4–5 and 10–11.**<br>Reported. Reference direction, units and denominators are checked. Derived crude log-Wald intervals are labelled separately; missing model CIs and target-population subgroups are not reconstructed.
Synthesis methods	13c	Describe any methods used to tabulate or visually display results of individual studies and syntheses.	**Sections 2.9 and 2.11, pp. 9–10; Table 1, pp. 13–15; Figures 2–3, pp. 19 and 21.**<br>Reported. Tables and study-specific plots distinguish measures and adjustment. Equal marker sizes are not weights; the pooled diamond is removed. ROBINS-E is displayed as recorded judgements.

Section / topic	Item	Checklist item	Location and reporting note
Synthesis methods	13d	Describe any methods used to synthesize results and provide a rationale for the choice(s). If meta-analysis was performed, describe the model(s), method(s) to identify the presence and extent of statistical heterogeneity, and software package(s) used.	**Section 2.11, pp. 9–10; SC-R2, Section K, pp. 10–11.** Reported for synthesis without meta-analysis. Clinical and methodological incompatibility explains non-pooling. The previous 180-minute pooled result was withdrawn; no pooled result is retained in this revision.
Synthesis methods	13e	Describe any methods used to explore possible causes of heterogeneity among study results (e.g. subgroup analysis, meta-regression).	**Sections 2.6–2.8 and 2.11, pp. 7–10.** Descriptive comparison reported by timing origin, population, outcome, surveillance and covariates. Formal interaction tests and meta-regression were not performed; contextual contrasts are not statistical subgroup analyses.
Synthesis methods	13f	Describe any sensitivity analyses conducted to assess robustness of the synthesized results.	**Sections 2.8 and 2.11, pp. 8–10; Section 3.7, pp. 19–20.** Not performed in the retained synthesis. Restricted evidence is described narratively; no leave-one-out, lower-risk, adjusted-only or long-term pooled sensitivity result is retained.
Reporting bias assessment	14	Describe any methods used to assess risk of bias due to missing results in a synthesis (arising from reporting biases).	**Section 2.12, p. 10; SC-R2, Section G, pp. 6–8.** Partly reported. Sparse groups and incompatible estimands precluded formal tests; data-derived cutoffs and incomplete analyses are identified as concerns. A complete synthesis-specific assessment is not documented.
Certainty assessment	15	Describe any methods used to assess certainty (or confidence) in the body of evidence for an outcome.	**Sections 2.4 and 2.10, pp. 6 and 9; SC-R2, Sections D and G.** Reported with a conduct limitation. GRADE is organised by comparison and time origin; observational evidence starts low. Single-study inconsistency and unestablished evidence bodies are qualified. Independent assessment was not completed.

Section / topic	Item	Checklist item	Location and reporting note
**RESULTS**			
Study selection	16a	Describe the results of the search and selection process, from the number of records identified in the search to the number of studies included in the review, ideally using a flow diagram.	**Section 3.1, pp. 10–11; Figure 1, p. 12; SC-R2, Section C, pp. 2–3.**<br>Partly reported. The 24 → 18 → 16 sequence is historical accounting, not a verified search-to-inclusion flow. Database yields, retrieval outcomes and final eligible-study counts are not established. The 36-entry candidate queue is not a PRISMA denominator.
Study selection	16b	Cite studies that might appear to meet the inclusion criteria, but which were excluded, and explain why they were excluded.	**Sections 3.1–3.2 and 3.4; Section 4.6, p. 23; SC-R2, Sections C and E.**<br>Partly reported. Leonidou and Al-Habsi have historical exclusion reasons; contextual and unresolved reports are qualified. A complete independently adjudicated full-text exclusion list is not available.
Study characteristics	17	Cite each included study and present its characteristics.	**Section 3.2 and Table 1, pp. 12–15; SC-R2, Section C, pp. 2–3.**<br>Reported for the historical inventory: 12 timing reports in Table 1 and four contextual reports in SC-R2. These are not 16 confirmed eligible adult-only studies; several applicability fields remain unresolved.
Risk of bias in studies	18	Present assessments of risk of bias for each included study.	**Section 3.8, p. 20; Figure 3, p. 21; SC-R2, Section F, p. 6.**<br>Recorded domain and overall assessments are displayed for 16 historical reports. These are not new independent appraisals; Thomas's rationale and design-specific tool applicability remain qualified.

Section / topic	Item	Checklist item	Location and reporting note
Results of individual studies	19	For all outcomes, present, for each study: (a) summary statistics for each group (where appropriate) and (b) an effect estimate and its precision (e.g. confidence/credible interval), ideally using structured tables or plots.	**Table 1, pp. 13–15; Section 3.6, pp. 16–18; Figure 2, p. 19; SC-R2, Sections E and K.** Partly reported. Available estimates, precision and aggregate cells are shown with source qualifications. Roddy's original CI and several group summaries remain unavailable. Unreported subgroup data and missing precision are not supplied.
Results of syntheses	20a	For each synthesis, briefly summarise the characteristics and risk of bias among contributing studies.	**Sections 3.2–3.8, pp. 12–20; SC-R2, Sections C, F and G.** Descriptive comparison-level summaries are reported, linking population, time origin, outcome, follow-up and bias concerns. Interpretation is limited to the preserved inventory and unverified appraisals.
Results of syntheses	20b	Present results of all statistical syntheses conducted. If meta-analysis was done, present for each the summary estimate and its precision (e.g. confidence/credible interval) and measures of statistical heterogeneity. If comparing groups, describe the direction of the effect.	**Section 2.11, pp. 9–10; Section 3.6, pp. 16–18; Figure 2, p. 19.** Not applicable to a retained statistical synthesis: none is reported. Withdrawal of the former 180-minute pool is explained. Current results are study-specific; no summary effect or heterogeneity statistic is retained.
Results of syntheses	20c	Present results of all investigations of possible causes of heterogeneity among study results.	**Sections 3.3–3.7, pp. 15–20; Table 1; Section 4.3, p. 22.** Descriptive sources of heterogeneity are reported. No formal subgroup interaction or meta-regression results are available; differences in significance are not treated as effect modification.
Results of syntheses	20d	Present results of all sensitivity analyses conducted to assess the robustness of the synthesized results.	**Section 3.7, pp. 19–20; Section 2.11, pp. 9–10.** Not applicable: no sensitivity analysis is retained after withdrawal of the pooled result. Contextual or restricted evidence descriptions are not presented as robustness tests.

Section / topic	Item	Checklist item	Location and reporting note
Reporting biases	21	Present assessments of risk of bias due to missing results (arising from reporting biases) for each synthesis assessed.	**Section 2.12, p. 10; Section 4.7, pp. 23–24; SC-R2, Section G, pp. 6–8.** No formal synthesis-specific assessment is reported. Sparse evidence and incomplete searching limit assessment; possible selective reporting is discussed without claiming that publication bias has been demonstrated.
Certainty of evidence	22	Present assessments of certainty (or confidence) in the body of evidence for each outcome assessed.	**Section 3.8, p. 20; SC-R2, Section G, pp. 6–8.** Qualified comparison-specific profiles are reported. Historical very-low ratings are not independently validated final assessments; no primary rating is assigned to unestablished target evidence bodies.
**DISCUSSION**			
Discussion	23a	Provide a general interpretation of the results in the context of other evidence.	**Sections 4.1–4.6, pp. 21–23; Conclusions, p. 24.** Reported. Findings are compared with previous reviews while separating process targets, causal thresholds and the limits of the preserved evidence.
Discussion	23b	Discuss any limitations of the evidence included in the review.	**Sections 4.1, 4.3–4.4 and 4.7, pp. 21–24.** Reported. Confounding, incompatible measures, mixed populations, uncertain exposure definitions, surveillance differences and imprecision are discussed.

Section / topic	Item	Checklist item	Location and reporting note
Discussion	23c	Discuss any limitations of the review processes used.	**Section 4.7, pp. 23–24; Abstract, p. 1; Sections 2.1, 2.3–2.5 and 2.9–2.10.**<br>Reported explicitly. Missing complete searches, non-independent selection/extraction/appraisal, retrospective registration and unquantified audit coverage are distinguished from primary-study bias.
Discussion	23d	Discuss implications of the results for practice, policy, and future research.	**Sections 4.2, 4.5 and 4.8, pp. 22–24; Conclusions, p. 24.**<br>Reported. Prompt prophylaxis, explicit first-dose/time-origin documentation, harmonised prospective outcomes and stronger review procedures are discussed without claiming a safe delay.
**OTHER INFORMATION**			
Registration and protocol	24a	Provide registration information for the review, including register name and registration number, or state that the review was not registered.	**Abstract, p. 1; Section 2.1, pp. 3–4; Data Availability Statement, p. 25; reference 17.**<br>Reported. OSF DOI 10.17605/OSF.IO/7J8QN is a retrospective registration dated 6 August 2026; prospective registration is not claimed.
Registration and protocol	24b	Indicate where the review protocol can be accessed, or state that a protocol was not prepared.	**Section 2.1, pp. 3–4; Data Availability Statement, p. 25; reference 17; SC-R2, Sections A and I.**<br>Access locations reported: retrospective OSF registration, associated project g3vma and preservation copy. The current revision is not claimed to be deposited in that project.

Section / topic	Item	Checklist item	Location and reporting note
Registration and protocol	24c	Describe and explain any amendments to information provided at registration or in the protocol.	**Sections 2.1, 2.6 and 2.11; Supplementary Materials, p. 25; SC-R2, Sections A and J.** Reported. Retrospectively documented categories, withdrawal of the pooled result and subsequent source/applicability corrections are distinguished from the original archive. The broader search amendment remains a draft.
Support	25	Describe sources of financial or non-financial support for the review, and the role of the funders or sponsors in the review.	**Funding and Acknowledgments, pp. 25–26.** Partly reported. No external funding is declared and AI assistance is described. Any other institutional/non-financial support or sponsor role is not explicitly specified.
Competing interests	26	Declare any competing interests of review authors.	**Conflicts of Interest, p. 26.** Reported. The manuscript states: “The authors declare no conflicts of interest.”
Availability of data, code and other materials	27	Report which of the following are publicly available and where they can be found: template data collection forms; data extracted from included studies; data used for all analyses; analytic code; any other materials used in the review.	**Supplementary Materials and Data Availability Statement, p. 25; SC-R2, Section I, p. 9.** Partly reported. The retrospective registration and local supporting materials are identified. Public deposition of the current revision is not verified; complete native exports and locked independent assessments are unavailable.

Source and licence

Page MJ, McKenzie JE, Bossuyt PM, Boutron I, Hoffmann TC, Mulrow CD, et al. The PRISMA 2020 statement: an updated guideline for reporting systematic reviews. BMJ. 2021;372:n71. doi:10.1136/bmj.n71.

Checklist wording reproduced under CC BY 4.0; layout and manuscript-specific reporting notes adapted. Official source: https://www.prisma-statement.org/prisma-2020-checklist. Licence: https://creativecommons.org/licenses/by/4.0/. No endorsement by the PRISMA authors is implied.
