# Supplementary material for: Timing of First Systemic Antibiotic Administration and Risk of Deep Infection in Adults with Open Long-Bone Fractures: A Systematic Review and Threshold-Specific Evidence Synthesis
Source: J Clin Med. 2026 Oct 4;15(19):7689. doi: 10.3390/jcm15197689 (PMC13665906; doi:10.3390/jcm15197689)
Supplement: Supplementary file 1 [file jcm-15-07689-s001.zip › jcm-4524900-supplementary.pdf]

Supplementary File SC-R2

Timing of First Systemic Antibiotic Administration and Risk of Deep Infection in Adults with Open Long-Bone Fractures: A Systematic Review and Threshold-Specific Evidence Synthesis

Manuscript ID: jcm-4524900 | 23 September 2026

Nguyen The Diep; Nguyen Minh Chau; Phan Thanh Nam; Tien Van Nguyen

Consolidated provenance, source corrections, and appraisal qualifications

This supplement reports the evidence available for the current revision. It does not certify a complete database rerun, independent duplicate review, or an OSF deposit. Numerical source corrections documented on 22 September 2026 are incorporated without changing the historical inclusion count or inventing subgroup data. Bracketed reference numbers correspond to the main manuscript.SA. Chronology and version precedence

Table S1. Chronology and version precedence.

Date / record	Documented activity	What it establishes
5 August 2026	Historical evidence-verification date in earlier versions.	Not a verified search date for every source.
6 August 2026	Retrospective OSF registration, DOI 10.17605/OSF.IO/7J8QN.	A retrospective archive; not preregistration.
10–17 September 2026	Failed acquisition requests and targeted source checks in earlier revision records.	No complete source search or independent assessment.
22 September 2026	Source re-extraction note: recovered printed intervals/precision and recorded inconsistencies.	Selected source corrections, not a full search yield.
23 September 2026, Round 2 package	Manuscript, response, supplementary record and figure-label consolidation.	Editorial/source integration; no new human review or public deposit.

The current SC-R2 supplement supersedes conflicting working statements about Hendrickson's unavailable intervals, Sulala's unresolved six-hour boundary, Kock's adult-only status, Roddy's complete 90-day follow-up, and automatic invalidity of Thomas's overall rating. The original registration and historical source files are not rewritten.

SB. Planned information sources and actual search status

The queries in the accompanying draft-strategy folder are preserved working strategies, not evidence of successful execution. Drafts without an age, language, or publication-date limit do not prove that no interface filter was applied in a historical search. The proposed two-concept amendment (open fracture AND antibacterial treatment, without mandatory timing or infection terms) remains a draft; its translation and host-specific validity have not been certified.

Table S2. Planned information sources and recorded search status.

Source	Status supported by the preserved record
MEDLINE/PubMed	Documented failed requests; no complete native result set.
Embase	No authenticated complete export established.
Scopus	No authenticated complete export established.
Web of Science Core Collection	No authenticated complete export established.
CENTRAL	No authenticated complete export established.
CINAHL	No authenticated complete export established.
Global Index Medicus	Failed request recorded in the 22 September source note.
ClinicalTrials.gov	Failed requests; no complete query/export established.
WHO ICTRP	Portal inspection recorded; no completed query/export established.
Europe PMC, supplementary	Failed requests; not a substitute for an unsearched planned source.

Successful execution dates, per-source result totals, complete exports, and a database-export deduplication record remain unavailable. None of these missing quantities is represented as zero. Targeted lookups of known articles, source PDFs, a query launcher, or a software test do not establish that a systematic search was completed.

SC. Inventory, reports, and Figure 1

The two preserved inventories contain 11 and 13 entries. The prior accounting records 24 entries, six entries set aside, 18 candidate reports, and 16 retained reports after two recorded exclusions. This establishes a historical accounting sequence, not independently confirmed retrieval, eligibility, or a count of unique eligible adult studies. The recorded reasons for setting aside Leonidou and Al-Habsi are retained as historical dispositions, not newly adjudicated exclusions. The supplied Figure 1 layout has been retained with corrected labels. Unverified zero-result/removal/retrieval labels and the “13 adult-only studies” claim are removed. The final box

refers to the historical map. The 36-entry targeted-verification queue described in the 22 September source note includes the 24 historical entries and 12 additional candidates, including reviews and unresolved reports. It is not a PRISMA denominator and is not added to Figure 1.

Table S3. Historical report inventory and analytic qualifications.

ID	Historical report	Analytic qualification
I01	Dellinger 1988	Contextual; age >14 years; minimum 21-day follow-up.
I02	Patzakis 1989	Contextual; mixed-age population; surveillance unclear.
I03	Al-Arabi 2007	Timing report; age, time origin, outcome and follow-up unresolved.
I04	Thomas 2013	Contextual; age 1–89; infection-or-nonunion endpoint.
I05	Zumsteg 2014	Timing report; adults; minimum follow-up/time origin unresolved.
I06	Lack 2015	Timing report; injury clock; 66-minute cutoff; 90-day infection.
I07	Johnson 2017	Contextual process comparison; not an exact timing-outcome contrast.
I08	Hendrickson 2020	Timing report; injury clock; coefficient scale unresolved.
I09	Roddy 2020	Narrative timing evidence; minimum 30 days; original CI unverified.
I10	Zuelzer 2021	Contextual for ≥90-day question; corrected 127-patient set.
I11	Grigorian 2023	Receiving-centre timing; inpatient composite outcome.
I12	Okhuereigbe 2025	Adults; adjusted ED-arrival timing results; facility status unresolved.
I13	Kock 2025	Contextual whole cohort; mixed ages and mechanisms.
I14	Haile 2025	Case-control timing evidence; design-appropriate appraisal unresolved.
I15	Morgan 2026	Adults; crude timing result; common outcome window unresolved.
I16	Sulala 2026	Contextual whole cohort; mixed ages and mechanisms.

These labels record current interpretation of the preserved reports, not new independent eligibility decisions. The 12 reports in main Table 1 plus the four contextual reports above account for the historical 16; placement in Table 1 is not certification of primary eligibility.

SD. Scope of the documented checks and independent-review status

The original selection was attributed to T.V.N. in the supplied manuscript. Subsequent checks compared identifiers, report relationships, outcome and population descriptions, model labels, numerical fields, and selected arithmetic against available passages. The coverage of those checks was not measured against an independent reference assessment. The A/B working forms supplied in earlier packages are uncompleted; they are not included here as evidence of completed review.

Table S4. Documented checks and independent-review status.

Domain	What is documented	What is not established
Eligibility	Historical dispositions and source-applicability notes.	Paired independent inclusion/exclusion decisions.
Time origin	Four-clock framework; prior doses and exact boundaries considered.	Independent classification of every study–effect record.
Numerical extraction	Selected source transcriptions and aggregate arithmetic checks.	A complete independently extracted paired dataset.
ROBINS-E	Recorded domain matrix and explicit uncertainty.	Independent signalling answers and consensus judgements.
GRADE	Qualified comparison-specific profiles.	Independent final ratings of a verified eligible evidence body.

No reviewer identity, date of completion, agreement percentage, kappa, accuracy statistic, or consensus decision has been created. Authorship is not a proxy for independent assessment. AI-assisted checking is identified separately and cannot supply the missing second-human assessment.

SE. Source-specific corrections and conservative use

SE.1. Roddy [32]

The primary abstract reports an adjusted Cox HR of 2.4 ($p = 0.036$), with adjustment for age, smoking and drug use, using a 120-minute ROC-derived threshold. Minimum follow-up was 30 days; the SSI endpoint was within 90 days. A later article cites a 95% CI of 1.1–5.7, while a publisher Discussion excerpt describes a 2.7-fold hazard. These do not establish the exact original-model interval or reconcile the model discrepancy. The point estimate remains qualified narrative evidence without an inferred CI bar, standard error or pooling weight. Missing precision alone is not a valid reason to invent an exclusion.

SE.2. Kock [26]

The report includes 227 patients with 237 fractures, ages 15–63 years, ten bilateral cases and 33 gunshot fractures. Minimum follow-up was three months after definitive management. The whole-cohort table gives 16 FRI/140 non-FRI below three hours and 10 FRI/71 non-FRI above three hours; published crude OR 1.2 (95% CI 0.5–2.9). These cells cannot recover a joint ≥18-year, nonballistic timing–FRI subgroup. Equality at three hours, earlier-facility dosing and bilateral-fracture handling remain unresolved. The whole-cohort result is contextual and is not relabelled as adult-only.

SE.3. Thomas [23]

The preserved source describes 138 transported cases across eight helicopter emergency medical service programmes, ages 1–89 years, and an infection-or-nonunion endpoint. Programme-specific treatment supports a confounding concern but does not establish every recorded domain judgement. ROBINS-E permits a justified cumulative upward overall judgement; this is not an automatic rule based on the number of high-risk domains. The historical very-high overall rationale remains unavailable. The score is retained as historical, not supplied with an invented original justification or used to rate an adult-only deep-infection comparison.

SE.4. Hendrickson [31]

The source note identifies accepted-manuscript PDF page 19, Table 2: within one hour, coefficient 0.1906 (95% CI −0.0363 to 0.4175; p = 0.099); within three hours, −0.0105 (−0.1188 to 0.0978; p = 0.848); continuous injury-to-antibiotic time, −0.0003 (−0.0010 to 0.0004; p = 0.431). The cohort contains 156 patients/159 fractures and 12 deep infections, with minimum one-year follow-up. Model terminology and table scale are not fully consistent. Values are transcribed as coefficients, not exponentiated as log ORs. Recovery of the intervals supersedes an absent-CI rationale but does not determine a new GRADE judgement.

SE.5. Morgan [36]

The overall early and later groups contain 34/270 and 29/207 deep infections. These cells agree between the source text and Table 2. The early anatomical subgroup events, however, sum to 38 rather than 34; the printed 6/66 subgroup cell is labelled 3.0%, although the fraction equals 9.09%. No anatomical subgroup result or corrected source count is inferred. Only the explicitly identified overall cells support the crude checking calculation.

SE.6. Sulala [27]

Publisher PDF pages 5–6, Tables 2–3, give ≥6 hours versus <6 hours and an exact printed aOR of 3.981 (95% CI 1.035–15.317; p = 0.044). The timing groups contain 56 later and 59 earlier patients, with ten and four FRI events. A printed crude-OR row (0.285; interval 0.84–0.970) is internally inconsistent and does not reproduce these cells. It has not been silently repaired. Ages

13–70 years, 82 bullet injuries and scheduled rather than complete minimum 12-month follow-up prevent assuming an eligible adult, nonballistic, long-term subgroup. The adjusted result is labelled as printed, not independently reproduced.

SE.7. Zuelzer, Leonidou, Al-Habsi, and companion reports

Zuelzer's linked erratum [18] distinguishes 142 initial patients/143 fractures from the 127-patient analysis set after insufficient-follow-up exclusions. It is one study, not a second cohort; minimum six-week follow-up limits the ≥90-day question. Leonidou's whole mixed-age cohort [25] separates deep from superficial events but not adult-only data. Al-Habsi's timing distribution among infected patients does not provide full exposure denominators for an infection-risk comparison. The Shannon conference/full-report relationship is unresolved; a related topic or DOI citation is not sufficient to establish a duplicate cohort.

SE.8. Targeted candidates outside the historical flow

The 22 September source note lists Weber, Enninghorst, Assunção, Tesso, Ukai, Brodell, Koulopoulos, Sheibani, Marten, Kadi, Ben Amar and Nakagawa as additional source-linked candidates, not twelve newly included studies. The Assunção timing cells concern infectious complications rather than verified isolated deep infection. No target subgroup or new inclusion total is inferred. These candidate dispositions require full eligibility review and are not evidence of a comprehensive search.

SF. Recorded ROBINS-E judgements

The following matrix reproduces the historical single-investigator judgements; no new appraisal has been performed. L = low; SC = some concerns; H = high; VH = very high. D1: confounding; D2: exposure measurement; D3: participant selection; D4: post-exposure interventions; D5: missing data; D6: outcome measurement; D7: reported-result selection.

Table S5. Recorded ROBINS-E judgements.

Report	D1	D2	D3	D4	D5	D6	D7	Overall
Dellinger 1988	H	H	SC	SC	H	H	SC	H
Patzakis 1989	VH	H	H	SC	H	H	H	VH
Al-Arabi 2007	VH	H	H	H	H	H	H	VH
Thomas 2013	H	SC	H	H	H	H	SC	VH
Zumsteg 2014	H	SC	SC	SC	H	SC	SC	H
Lack 2015	H	SC	SC	SC	SC	SC	H	H
Johnson 2017	H	SC	SC	H	H	H	SC	H
Hendrickson 2020	H	SC	SC	SC	SC	SC	SC	H
Roddy 2020	H	SC	SC	SC	SC	SC	H	H

Report	D1	D2	D3	D4	D5	D6	D7	Overall
Zuelzer 2021	H	H	H	H	H	SC	H	H
Grigorian 2023	H	SC	SC	SC	H	H	H	H
Okhuereigbe 2025	SC	L	SC	SC	SC	L	SC	SC
Kock 2025	H	L	SC	SC	SC	L	SC	H
Haile 2025	H	SC	H	SC	SC	L	H	H
Morgan 2026	H	SC	SC	SC	H	SC	SC	H
Sulala 2026	H	SC	SC	H	H	SC	SC	H

Totals are one SC, twelve H and three VH overall; fifteen D1 ratings are H/VH. Recounting the categories does not validate them. Thomas's cumulative rationale is unavailable. The tool's follow-up-study scope does not automatically validate its historical use for Haile's case-control design. These qualifications apply to Figure 3 as well as the matrix.

SG. Qualified GRADE profiles

These are carried-forward comparison-specific evidence descriptions, not final independent GRADE ratings. Observational evidence was initially rated low. Sparse or single-study groups are not automatically downgraded for inconsistency, and publication bias is not demonstrated by the limited number of studies. No common absolute effect or NNT is calculated from incompatible populations and baseline risks. No primary rating is assigned where the target eligible evidence body has not been established.

60 min | injury

Hendrickson: coefficient 0.1906 (−0.0363 to 0.4175).

Historical very low. Confounding, selected GA IIIB tibia population and model-scale ambiguity limit inference. The recovered CI replaces missing-CI reasoning; no new rating assigned.

60 min | hospital/ED

Okhuereigbe: aOR 1.02 (0.39–2.68).

Historical very low. Residual confounding, selected tibia population, unresolved facility position and wide precision interval. Inconsistency not assessable for one study.

60 min | receiving centre

Grigorian: adjusted ratio unavailable; Morgan: crude OR 1.13 (0.66–1.93).

Historical very low; not a pooled evidence body. Inpatient composite versus unclear common deep-infection follow-up, different adjustment, and residual confounding.

120 min | hospital/ED

Roddy: abstract aHR 2.4; original CI unverified.

No final primary rating. Minimum 30-day follow-up differs from the 90-day endpoint; precision and model attribution unresolved; ROC-derived cutoff.

180 min | injury

Hendrickson: coefficient −0.0105 (−0.1188 to 0.0978).

Historical very low with the same model/applicability concerns. This is the same cohort as the one-hour contrast, not independent replication.

180 min | hospital/ED

Okhuereigbe: aOR 1.08 (0.35–3.37).

Historical very low. Confounding, selected population, uncertain facility position and wide interval. Not combined with Kock's crude whole-cohort result.

3 h | mixed-age hospital cohort

Kock: crude OR 1.2 (0.5–2.9).

No adult-only primary rating; target subgroup unreported. Contextual mixed ages/mechanisms and unresolved equality at the cutoff.

66 min | injury

Lack: aOR 3.78 (1.16–12.31).

Historical very low. Confounding, study-specific cutoff selection, selected severe tibia population and wide interval; no causal threshold established.

150 min | injury

Zuelzer: OR 5.60 (1.40–22.20); adjustment attribution unresolved.

Contextual for the minimum-90-day question because follow-up was at least six weeks.

Historical very low; corrected analysis denominator is not independent validation.

≥6 h versus <6 h | injury

Sulala: printed aOR 3.981 (1.035–15.317).

No adult-only primary rating. Whole-cohort mixed ages/mechanisms, ascertainment and crude-row inconsistencies; original adjusted model not reproduced.

3–12 h and >12 h | injury

Haile: aOR 1.16 (0.35–4.13) and 4.92 (1.63–16.90), reference <3 h.

Historical very low. Design-appropriate risk-of-bias assessment required; selected case-control setting and wide intervals. Contrasts are correlated.

Consensus FRI AND minimum 12 months

No confirmed common eligible evidence body.

Not rated. Minimum follow-up, scheduled surveillance and strict consensus FRI are not interchangeable.

SH. Causal and time-origin framework

The retrospectively documented framework separates injury-to-first-dose, first-medical-contact-to-first-dose, first-hospital-arrival-to-first-dose, and receiving-centre-arrival-to-first-dose intervals. A dose given before transfer remains the first dose. Baseline injury, host and system factors can influence timing and infection. Later debridement, fixation or coverage requires an explicit causal interpretation before adjustment; occurrence after antibiotics alone does not prove mediation or inappropriate adjustment. The framework aids interpretation but does not demonstrate control of confounding.

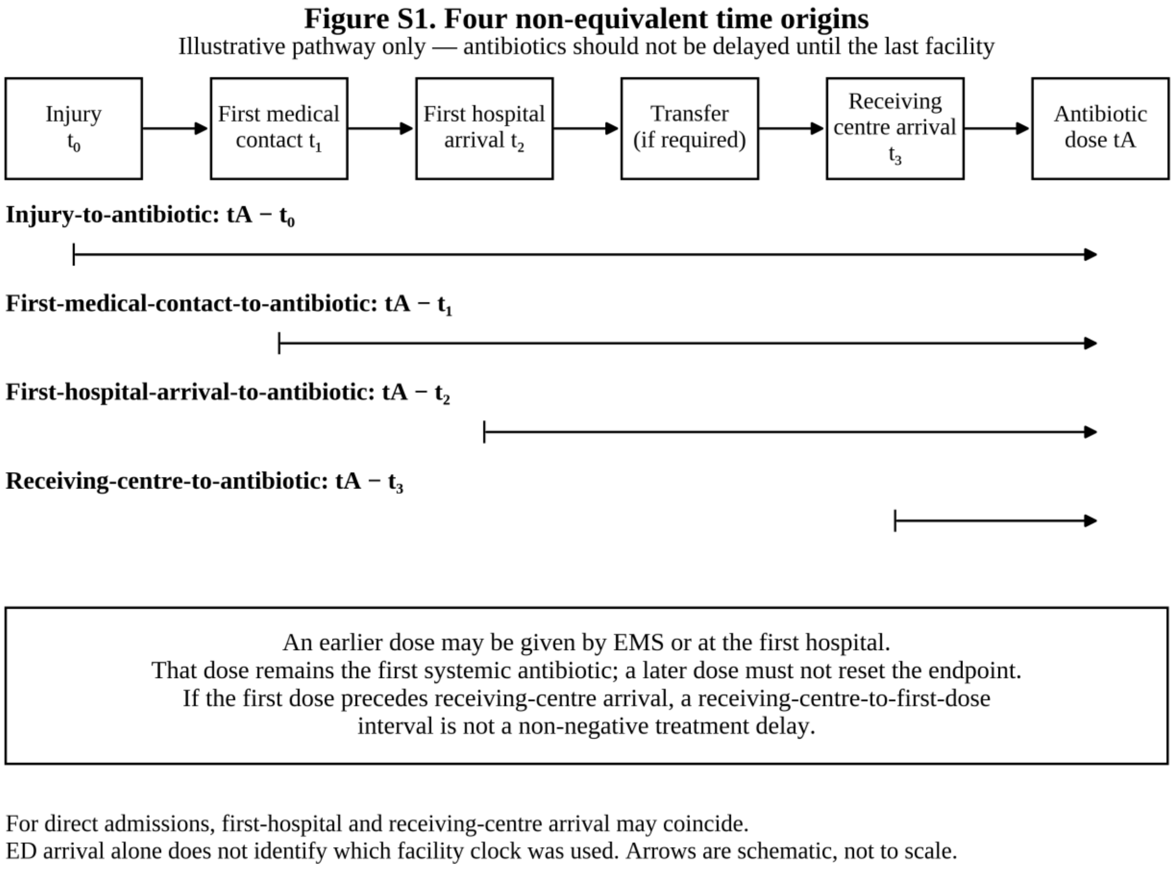

Figure S1. Distinct time origins and the true first-dose endpoint. Retained from the earlier revision; no additional clinical result is implied.

SI. Availability, permissions and limitations

The original OSF DOI remains 10.17605/OSF.IO/7J8QN, associated project g3vma. The registration is retrospective and identifies CC0 1.0 Universal. No upload of the present package or renewed public-access check is claimed. Original copyrighted article PDFs and private correspondence are excluded. Locally supplied evidence summaries do not imply that all planned database searches or independent assessments were completed.

SJ. Round 2 changes and reporting map

Table S6. Round 2 changes and reporting map.

**Reviewer issue**	**Locations in the revised manuscript**	**Implemented change**
Search provenance	Abstract; 2.1, 2.3; 3.1; 4.7; Figure 1	Historical inventory separated from completed searches and final eligibility; unavailable yields not zero.
Independent assessment	Abstract; 2.4–2.5; 2.9–2.10; 3.8; 4.7	Actual single-investigator process stated; no fabricated assessor, consensus or accuracy statistic.
Source precision and applicability	Table 1; 2.5, 2.11; 3.6; Figure 2	Recovered Hendrickson/Sulala values; Roddy, Kock and Thomas limitations retained.
Clinical interpretation	4.1–4.7; Conclusions	Study-specific, bounded interpretation; no restored pool, no causal or safe-delay claim.
Repository/materials	Supplementary Materials; Data Availability	Consolidated local package identified; no false deposition statement.

PRISMA reporting items concerning sources/search strategies (6–7), selection and extraction (8–9), final flow and exclusions (16a–16b), and availability (27) remain incompletely supported as conduct requirements. A reporting map is not a certificate of completed systematic-review methods. Findings and appraisals in items 17–22 remain subject to the stated evidence-set and independent-review limitations.

SK. Inventory reconciliation, checking calculations and analytic sequence

Original source rows remain unchanged. Working identifiers link each inventory entry to its report, study relationship and recorded disposition. Matching uses reliable DOI/PMID plus title, authors, year and publication type; abstracts, posters, full reports, and corrections require a separate companion-report decision. No native-export deduplication total is inferred from the two inventory files. The sequential interpretation is:

population/anatomy/mechanism/outcome/follow-up applicability; true first dose and time origin; exact cutoff/reference; effect measure and adjustment; cohort/report linkage; study-specific presentation without pooling.

Table S7. Aggregate arithmetic checks and their limitations.

Checking input (later vs earlier)	Derived crude OR (95% log-Wald CI)	Boundary
Morgan: 29/207 vs 34/270	1.131 (0.664–1.926)	Overall deep infection only; not adjusted.
Kock: 10/81 vs 16/156	1.232 (0.532–2.855)	Whole fracture cohort; no clustering or adult-only adjustment.
Sulala: 10/56 vs 4/59	2.989 (0.879–10.164)	Whole mixed cohort; not a repair of the source crude-OR row.
Leonidou: 2/32 vs 5/129	1.653 (0.306–8.939)	Deep endpoint in mixed-age cohort; non-deep includes superficial events.
Assunção: 18/69 vs 27/172	1.895 (0.964–3.728)	Infectious-complication endpoint, not isolated deep infection.

For checking only: OR = a×d/(b×c), where a and b are later-group events/non-events and c and d are earlier-group events/non-events; SE[log(OR)] = $\sqrt{1/a + 1/b + 1/c + 1/d}$. These are derived intervals, not substituted source-reported model intervals. Calculations do not certify independent extraction, cohort eligibility or a causal effect.

Proof-stage update (3 October 2026): section and table labels and manuscript-reference numbers have been synchronised with manuscript.v9. The Zuelzer article and erratum share one reference entry. No study count, numerical effect, ROBINS-E judgement, GRADE rating, or search/independent-review status was changed by these editorial updates.

Provenance: current uploaded manuscript jcm-4524900(3).docx; preserved Supplementary File SC (10 September 2026); Supplementary Note R1-EX (22 September 2026), Sections SD–SH, with source page/table locators. The main manuscript retains references [13–17,20,21,34] for reporting/appraisal and [18–38] for the source reports. No new original-study dataset or author reply was obtained during this editorial consolidation.
